# Targeting the PI3K/AKT/mTOR and RAF/MEK/ERK pathways for cancer therapy

**DOI:** 10.1186/s43556-022-00110-2

**Published:** 2022-12-21

**Authors:** Qingfang Li, Zhihui Li, Ting Luo, Huashan Shi

**Affiliations:** 1 Laboratory of Aging Research and Cancer Drug Target, State Key Laboratory of Biotherapy, West China Hospital, National Clinical Research Center for Geriatrics, Sichuan University, Chengdu, China; 2 Department of Oncology, The General Hospital of Western Theater Command, Chengdu, PR China; 3 Department of Breast, Cancer Center, West China Hospital, Sichuan University, 610041 Chengdu, P. R. China; 4 Department of Biotherapy, Cancer Center, West China Hospital, Sichuan University, 610041 Chengdu, P. R. China

**Keywords:** PI3K/AKT/mTOR, RAF/MEK/ERK, Small molecular inhibitors, Cancer therapy, Combination inhibitor

## Abstract

The PI3K/AKT/mTOR and RAF/MEK/ERK pathways are commonly activated by mutations and chromosomal translocation in vital targets. The PI3K/AKT/mTOR signaling pathway is dysregulated in nearly all kinds of neoplasms, with the component in this pathway alternations. RAF/MEK/ERK signaling cascades are used to conduct signaling from the cell surface to the nucleus to mediate gene expression, cell cycle processes and apoptosis. RAS, B-Raf, PI3K, and PTEN are frequent upstream alternative sites. These mutations resulted in activated cell growth and downregulated cell apoptosis. The two pathways interact with each other to participate in tumorigenesis. PTEN alterations suppress RAF/MEK/ERK pathway activity via AKT phosphorylation and RAS inhibition. Several inhibitors targeting major components of these two pathways have been supported by the FDA. Dozens of agents in these two pathways have attracted great attention and have been assessed in clinical trials. The combination of small molecular inhibitors with traditional regimens has also been explored. Furthermore, dual inhibitors provide new insight into antitumor activity. This review will further comprehensively describe the genetic alterations in normal patients and tumor patients and discuss the role of targeted inhibitors in malignant neoplasm therapy. We hope this review will promote a comprehensive understanding of the role of the PI3K/AKT/mTOR and RAF/MEK/ERK signaling pathways in facilitating tumors and will help direct drug selection for tumor therapy.

## Introduction

**Fig. 1:**
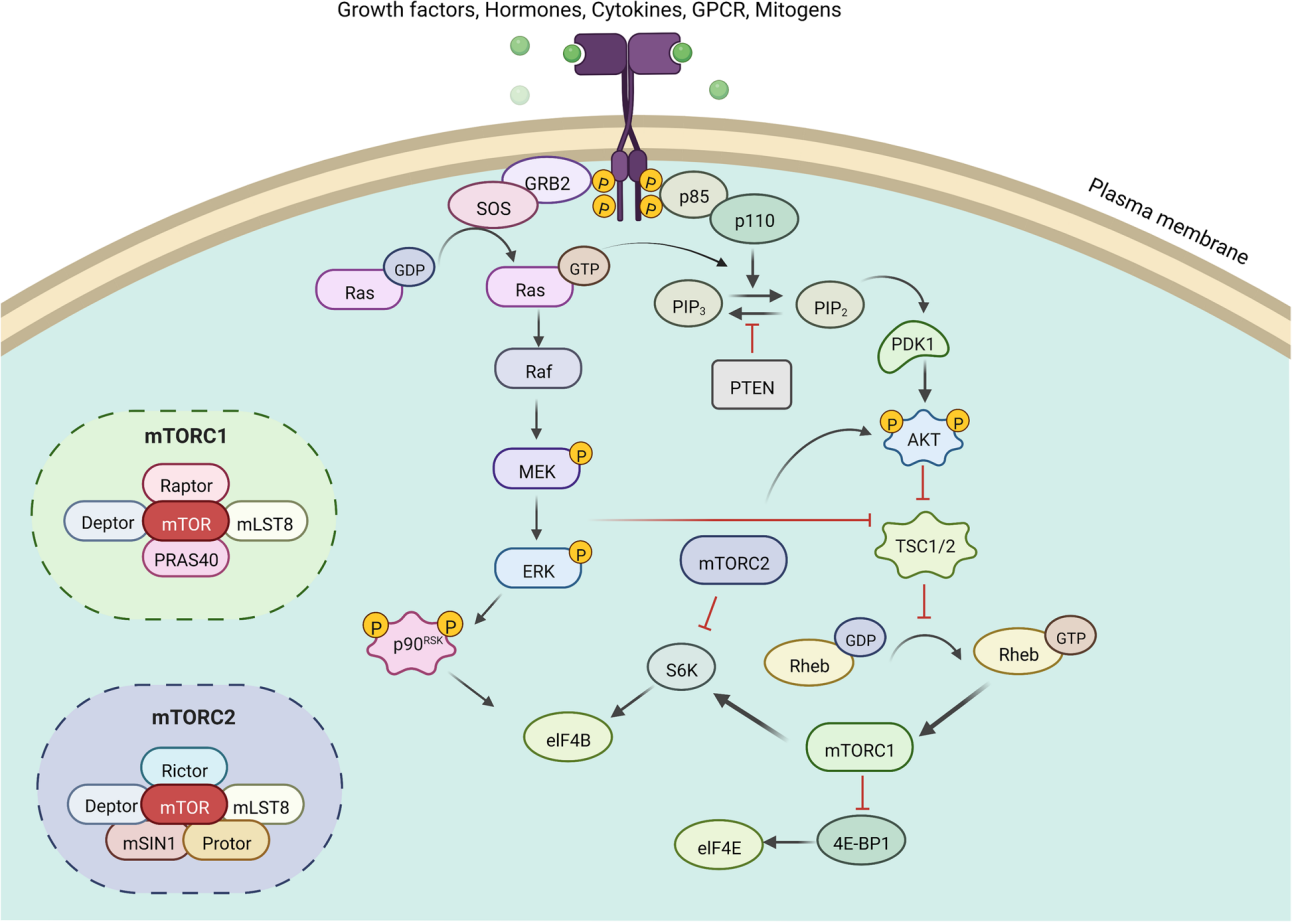
Schematic of the PI3K/AKT/mTOR and Raf/MEK/ERK signaling pathways. Growth factors, hormones, cytokines, GPCRs, and mitogens activate receptor tyrosine kinases (RTKs) recruiting PI3K to attach to the plasma membrane, where PI3K catalyzes PI (4,5) P2 to PI (3,4,5) P3. PTEN suppressed the process, and PTEN mutations could induce abnormal activation. PI (3,4,5) P3 promotes AKT activation via the activity of PDK1 and mTORC2. AKT activation induced cell cycle progression, cell growth, cell apoptosis, survival, glucose metabolism, protein synthesis, signal triggering and transduction, and phosphorylation of the downstream substrate TSC2. AKT activation suppressed the activity of TSC2 to promote the production of Rheb complex, resulting in mTORC1 activation. mTORC1 activation facilitates the initiation of eukaryotic protein translation. 4E-binding protein 1 (4E-BP1) activation enhanced the release of eukaryotic translation initiation factor 4E (eIF4E). RTK activation further accelerates guanine exchange factor to load RAS with GTP. RAS–GTP dimers recruit RAFs or RAF/MEK heterodimers to membranes, where tetramers consisting of RAF and MEK promote RAF activation. MEK activation is initiated by docking on RAF dimers, which further facilitate ERK phosphorylation. RTKs, receptor tyrosine kinase; PI3K, phosphatidylinositol 3-kinase; PIP2, phosphatidylinositol 4,5-bisphosphate; PIP3, phosphatidylinositol 3,4,5-triphosphate; PTEN, phosphatase and tensin homolog; PDK1,3-pphosphoinositide-dependent kinase 1; mTOR, mechanistic target of rapamycin; TSC1, tuberous sclerosis 1; TSC2, tuberous sclerosis 2; 4E-BP, eIF4E-binding protein; GRB10, growth factor receptor-bound protein 10; IGF-1R, insulin-like growth factor 1 receptor; mLST8, mammalian lethal with SEC thirteen 8; RICTOR, rapamycin-insensitive companion of mTOR; S6K, ribosomal S6 kinase; FLCN, folliculin; ULK1, UNC-51-like kinase 1; RAPTOR, regulatory-associated protein of mTOR; RICTOR, rapamycin-insensitive companion of mTOR.

With the discovery of oncogenes and antioncogenes in the 1980s, the novel targeted treatment of imatinib was first approved in 2001 by the FDA [1–3]. The requirement of new anticancer medicines to the molecular groundwork is significantly higher than that of traditional chemotherapeutic drugs, of which their molecular mechanism of action was not identified empirically [3]. Kinases and phosphatases have key roles in controlling cellular functions [4]. There are currently seventy-one kinase inhibitors approved by the Food and Drug Administration (FDA) and 16 attached inhibitors approved by other countries and regions’ regulatory agencies [5]. The PI3K/AKT/mTOR and RAF/MEK/ERK signaling pathways are composed of kinase cascades that are managed by phosphorylation and dephosphorylation by particular kinases, phosphatases, and proteins regulating the exchange [6] (Fig. 1). The PI3K/AKT/mTOR and RAF/MEK/ERK signaling pathways have been extensively studied over the past 25 years [7]. Enormous breakthroughs in component detection and the mechanisms of how the components relay on the signals and mutations result in aberrant signaling and uncontrolled proliferation diseases. Broader perspectives and feedback loops have been identified, leading to more choice in blocking the pathways.

PI3K/AKT/mTOR signaling is a crucial intracellular pathway in regulating fundamental cellular functions, including but not limited to regulating cell growth, motility, survival, metabolism, and angiogenesis. Hyperactivation of the PI3K/AKT/mTOR pathway occurs in nearly all malignant neoplasms [8]. A previous study showed that single nucleotide polymorphisms (SNPs) in the PI3K/AKT/mTOR pathway are connected to the distant metastasis of carcinomas [8]. This pathway also has a vital role in promoting cell apoptosis through inhibition of related genes such as p53, caspase 3, Fas receptor (CD95) and TNF receptor (TNFR1) [9, 10].

AKT could serve as the targeted effector to the cell surface for activation. Two sites (T308 and S473) of AKT could be phosphorylated. In addition to phosphotidylinositide-dependent kinases (PDKs), AKT can be phosphorylated by mTOR. This signaling activation contributed to the growth of cells by suppressing autophagy via the activation of mechanistic target of rapamycin (mTOR) [11, 12]. MTOR can inhibit the initiation of autophagy via distinct usual pathways [13, 14]. In addition, PI3K/AKT/mTOR lead to epithelial-mesenchymal transition (EMT) in chemotherapy resistance and metastasis in malignant tumor cells [15–17].

The RAF/MEK/ERK pathway is a critical signaling pathway in transmitting signals from membranes to the nucleus [18]. The RAF family serine/threonine (ser/thr) protein kinases (Raf-1(C-Raf), B-Raf, A-Raf) activate MEK by phosphorylation, and MEK phosphorylation promotes ERK 1 and 2 phosphorylation at their residues. RAS GTPases and growth factor receptors, such as epidermal growth factor receptor (EGFR), control the activation of the RAF/MEK/ERK signaling pathway. Similar to PI3K/AKT/mTOR, activation of RAF/MEK/ERK signaling pathways was observed in a large fraction of solid cancers [6]. These observations contribute to the development of inhibitors targeting kinases containing the new RAF and MEK kinases approved by the FDA. Extracellular signal-regulated kinases (ERK1/2) are a subfamily of mitogen-activated protein kinases (MAPKs) that facilitate the culmination of signal transduction and regulate transcription. ERK1/2 are phosphorylated by the upstream MAPK/ERK kinases MEK1/2, which are tyrosine/threonine protein kinases that are necessary for proliferation and regular growth in human cells.

Both pathways share common inputs and can also be activated via RAS. In addition, when one pathway is suppressed, the other pathway may offer compensatory effectiveness [19]. MTOR, a downstream molecule phosphorylated by AKT, is inhibited, and PI3K can stimulate MAPK through RAS. These results showed that these two pathways serve as a complex network and provide a method for dual therapeutic compounds that can simultaneously block both pathways [20]. In addition to mTOR, several nodes of the PI3K/AKT/mTOR and RAF/MEK/ERK pathways are well known to interact with each other, and mounting evidence indicates that dual blockade of both pathways might contribute to anticancer effects [21]. Mutations observed in the genes of the pathway or in upstream receptors that activate these pathways [22]. The particular combination of phosphoinositide 3-kinase or phosphatidylinositol-3 kinase (PI3K) and MEK inhibitors is generally being evaluated in several clinical studies in various kinds of cancers.

PI3K/AKT/mTOR and RAF/MEK/ERK contain various kinases regulated by phosphorylation and dephosphorylation via relevant kinases. These two signaling pathways are considered vital oncogenic signaling pathways in tumorigenesis. In the current review, we describe the critical role of the PI3K/AKT/mTOR and RAF/MEK/ERK signaling pathways in carcinoma initiation and tumor development as potential strategies for tumor therapy, summarizing the recent doubts about targeting the pathway in monotherapy and combination therapy.

### Overview of the PI3K/AKT/mTOR pathway and its role

The signaling pathway composed of PI3K, protein kinase B (AKT), and mTOR is a part of a complicated signaling cascade comprising distinct upstream regulators and downstream effectors, which play critical roles in the formation processes of human cancers [23]. Prior evidence has identified that hyperactivation of PI3K/AKT/mTOR signaling promotes tumorigenesis. PI3K was first identified as a lipid kinase in the 1980s in the Cantley group’s study. Furthermore, the first clone and report of TOR was completed in 1991 by Hall, and its mammalian homolog mTOR was developed three years later [24].

Vital mouse models serving as key genetic evidence to identify the imperative roles of the PI3K/AKT/mTOR signaling pathway in promoting tumorigenesis have been constructed in recent years, contributing to the comprehension of the signaling by the recognition of whole modules via various methods and supplying valuable information for patients to confirm activation of the pathways in human carcinomas by deep sequencing, proteomics, reversed-phase protein arrays (RPPA) and bioinformatics approaches [25]. Different therapeutic agents targeting different components in the PI3K/AKT/mTOR signaling pathway have been designed and applied as anticancer agents [26].

PI3Ks promoted the transfer from PIP2 to PIP3 via phosphorylation of phosphatidylinositol. PIP3 is the basis of multiple downstream targets of the PI3K/AKT/mTOR pathway [27]. PI3Ks contain three classes: PI3Ks class I, PI3Ks class II and PI3Ks class III, which are separated by their structure, reaction mechanism, and characteristic [28–31]. Both Class IA and Class IB can be generally activated by G protein-coupled receptors (GPCRs), either via Gβγ protein or indirectly via RAS [32]. In addition, Class IA could also be regulated by receptor tyrosine kinases (RTKs) and upstream oncogenes. Class IA PI3Ks contain a regulatory subunit (p85α, p85β, p85γ) and a catalytic unit (p110α, p110β, p110δ, p110γ), which belong to heterodimers [29, 33] (Table 1). The regulatory subunit can be activated by the catalytic subunit [32]. After stimulation or subsequent activation, class IA PI3Ks can be recruited to the cytomembrane via the p85 subunit for motif phosphorylation. The activation of the p110 catalytic subunit in turn activated downstream signals [34]. P85 combined with the receptors, and p110 catalyzed the formation of PIP3 by adding an additional phosphate on PIP2. PTEN promoted the transfer of PI (3,4,5) P3 back to PI (4,5) P2 to reduce PIP3 by intrinsic lipid phosphatase. The N-terminal region of AKT docked to PI (3,4,5) P3 contributes to the translocation to the cytomembrane, resulting in AKT activation with two vital amino acid residues phosphorylated [35]. AKT induced the phosphorylation of PRAS40 and tuberous sclerosis complex (TSC2) to alleviate the inhibitory effect on mTORC1 to induce mTOR activation. In addition to mTORC1, mTORC2 is another complex of mTOR. MTOR is a kind of ser/thr protein kinase in PI3Ks kinases [36]. The Ragulator/Rag GTPase complex acts as a regulator in controlling mTORC1 activity. S6K1 and 4E-BP1 are downstream effectors of mTOR, which mediates protein synthesis [37]. AKT is a key node that transduces signals from mTORC2 to mTORC1, while mTORC1 could be regulated independently of mTORC2. MTORC1 activation by amino acids is mediated by Ras-related GTP binding (RAG) GTPases. Amino acids activate mTORC1 via Rag GTPases, which are recruited to lysosomes by the Ragulator complex (MAPK and TOR activator). Downregulated cellular energy promotes AMPK activation to trigger Raptor phosphorylation to inhibit mTORC1 action [38]. MTORC1 activation was promoted by ERK-dependent Raptor phosphorylation by RAS/MAPK activation. More alternations occurring in this pathway according to transcription, protein production, and other factors were discussed in next paragraph.

**Table 1:** Different classes of PI3K enzymes and their functions

PI3K class		Subunit	Gene	Protein	Aliases	Cellular functions
Class I	IA	Catalytic	PIK3CA	PI3K, catalytic, α polypeptide	p110α	Integrates extracellular signals from insulin and growth factors together with energy status, oxygenation and nutrient availability to modulate processes including cell growth, survival, proliferation, glucose metabolism, and angiognesis
			PIK3CB	PI3K, catalytic, β polypeptide	p110β	
			PIK3CD	PI3K, catalytic, δ polypeptide	p110δ	
		Regulatory	PIK3R1	PI3K, regulatory subunit 1 (α)	p85α	
			PIK3R2	PI3K, regulatory subunit 2 (β)	p85β	
			PIK3R3	PI3K, regulatory subunit 3 (γ)	p55γ	
	IB	Catalytic	PI3K3CG	PI3K, catalytic, γ polypeptide	p110γ	
		Regulatory	PIK3R5	PI3K, regulatory subunit 5	p101	
			PIK3R6	PI3K, regulatory subunit 6	p87/p84	
Class II		Catalytic	PIK3C2A	PI3K, class 2, α polypeptide	PI3K-C2α	Exocytosis, promote insulin secretion and neurosecretory granules release, regulate glucose transport, endocytosis, activation of Rho GTPases in cell contraction and migration.
			PIK3C2B	PI3K, class 2, β polypeptide	PI3K-C2β	
			PIK3C2G	PI3K, class 2, γ polypeptide	PI3K-C2ϒ	
Class III		Catalytic	PIK3C3	PI3K, class 3	Vps34	Endosome maturation, endosomal protein sorting, autophagosome formation and autophagy flux, cytokinesis.
		Regulatory	PIK3R4	PI3K, regulatory subunit 4	Vps15/ p150	

#### Dysregulation of the PI3K/AKT/mTOR pathway in neoplasms mediated by genetic alterations

##### Gene mutagenesis-induced overactivation of the PI3K/AKT/mTOR pathway in neoplasms

Referencing previous studies, the frequency of the PIK3CA mutated gene varies from 10 to 15% in human cancers [39]. PIK3CA mutations are found in nearly all neoplasm types, such as mammary tumors, colorectal carcinoma, esophageal cancer, gallbladder carcinoma, non-small cell lung cancer (NSCLC), ovarian cancer, and gastric carcinoma [40]. PIK3CA mutations in breast carcinoma are the most prevalent. H1047R, E545K, E542K, N345K, and H1047 L were the top five mutations that accounted for three-quarters of all PIK3CA mutations [41]. PIK3CA gene amplification is frequent in gastric carcinoma (36.4%), thyroid adenocarcinoma (30%), prostatic cancer (28%), ovarian cancer (13.3–29.8%), and cervical carcinoma (9.0–80%). Various positions were observed to be mutated in the PIK3CB gene, including lung carcinoma, thyroid cancer, and lymphoma [42]. These studies implied that the function of the PI3K isoform was extremely different. Clinically, upregulated PIK3CA expression was significantly related to neoplasm invasiveness, poor patient survival and lymph node metastasis [43].

AKT is a ser/thr kinase in downstream effectors of the PI3K/AKT/mTOR signaling pathway and includes three subtypes: AKT1, AKT2 and AKT3 [44]. AKT1 is observed in the majority of tissues, AKT2 is mainly found in organisms with high sensitivity to insulin, and AKT3 is expressed in the brain and testicles [45, 46]. PI3K facilitates Akt1 in cytoplasm transport to interact with PIP3 on the cytomembrane, leading to Akt1 phosphorylation and activation [47]. In addition, negatively regulated PI3K activation can inhibit Akt1 via PTEN phosphorylation. In conclusion, positive regulation of PI3K signaling and negative regulation of PTEN signaling can induce Akt1 activation in human cancers. Akt1 facilitates cell proliferation, survival, and metabolism via its downstream effectors BAD, FOXO1, and TSC1/2 [9]. Genomic alterations of Akt1 could directly promote the activation of Akt1 without phosphoinositide.

Mutations in Akt2 and Akt3 disrupt the role of pleckstrin homology (PH) and kinase domain (KD), resulting in AKT oncogenic activation [48]. Akt1 amplification is frequently reported to promote cisplatin resistance in epithelial cancers, such as gastric carcinoma, breast neoplasm, gallbladder tumor, NSCLC, and SCLC [49]. Akt2 gene copy number gain was found in ovarian cancer, pancreatic carcinoma, liver cancer, colorectal carcinoma, gastric carcinoma and breast neoplasm [50]. Akt3 gene alterations are rare in carcinomas. The Akt1-E17K mutation existing in the PH domain has been observed frequently in breast carcinoma, ovarian carcinoma, and malignant meningioma with increased oncogenicity, particularly by facilitating AKT binding with PIP3, which promotes the action of AKT [51]. The The Akt1-E17K mutation in mouse models contributed to mammary hyperplasia and resulted in lung epithelium disorder, demonstrating that Akt-activating mutation plays an oncogenic role in promoting tumorigenesis [52]. The other two subtypes of AKT-E17K mutations were less frequent than The Akt1-E17K mutations. The Akt2-E17K mutation is commonly observed in hyperinsulin mucoglycemia with dysregulated insulin production [53]. The Akt3-E17K somatic mutation was observed in human malignant melanoma [52]. Mutations in kinases manage the development of tumors and developmental disorders by promoting kinase activation [54].

The mTOR activation mutations increase its kinase activity, contributing to overactive downstream pro-proliferation signaling pathways [55]. The mutation rate in metastatic cancer was 3% (329/10,336) in the MSK IMPACT Clinical Sequencing Cohort, which is similar to that (2.9%, 292/10,194) of the China Pancancer Cohort (OrigiMed2020). The rate of nonsynonymous mTOR mutations was approximately 10% in malignant melanoma patients, and nonsynonymous mTOR mutations were connected to a poor prognosis [56]. The mTOR mutation was found in a wide variety of malignant tumors, including lung, renal cell, endometrium, colorectal and squamous carcinoma [57]. HEAT repeat, FAT domain, and KD mutations promote the oncogenic role of mTOR in tumorigenesis [58]. Rictor and mTOR are prevalently observed in cancer, while mutations in mSin1, mLST8 and Raptor are not common in human cancers [59]. Rictor amplification is a selection criterion for potential mTOR inhibition treatment.

##### Deletion of genes promotes hyperactivity of the PI3K/AKT/mTOR pathway

PTEN, the most common tumor suppressor gene, is the major PI(3,4,5)P3 kinase that antagonizes PI3K phosphorylation, and its loss could contribute to uncontrolled PI3K signal transduction [60, 61]. PTEN deletion in different neoplasms has been found in either genetic or epigenetic mechanisms [62–64]. Moreover, the deletion of PTEN leads to its loss efficiency and is closely correlated with worse prognosis, drug resistance and advanced tumor stages. PTEN deletion is frequently found in solid tumors and hematologic malignancies, including prostate cancer, diffuse large B-cell lymphoma, glioma, endometrial carcinoma, hepatopancreatic ductal malignancy, and invasive bladder cancer [65–71]. PTEN neddylation is promoted by Nedd8 interaction at high glucose levels, which does not lead to an increase in PTEN accumulation in the nucleus [72]. PTEN in the nucleus could promote RAD51 expression, which results in DNA double-strand breaks.

PTEN mutations plays an important role in PI3K pathway activation in tumor occurrence and contributes to a majority of activates in tumor prognosis [73]. PTEN mutations presumably lead to the hyperactivation of all PI3K-mediated pathways. However, PTEN mutations were not observed in some neoplasms. In head and neck cancers, 15% of PTEN mutations was found [74]. In breast carcinomas, only 3% of PTEN mutations was identified [73]. It is clear that some other signaling pathway existed besides PTEN mutations, activation and deletions to constitutively activated PI3K activity. The interaction between RAS and PI3K was supported by the vivo data. However, RAS and PTEN mutations is mutually exclusive in neoplasm of endometrium and malignant melanomas. PTEN mutations were commonly observed in spongioblastoma but rarely seen in carcinoma of pancreas, lung and colorectum [75–80]. The mutations of RAS are opposite in these neoplasms. Compared with Pten^+/−^ mice, RAS mutations was commonly observed in Pten^+/+^ mice in according to chemically induced skin neoplasms. Lacking RAS mutations results in second Pten allele loss. The results demonstrated RAS and PTEN may have synergistic effect in carcinogenesis.

PTEN promoter methylation induced PTEN transcription reduction that correlated with the relapse and recurrence of gastric cancer via PI3K/AKT/mTOR signaling pathway hyperactivation [81–83]. Increased PTEN promoter methylation could decrease PTEN transcription, resulting in worse survival [82]. P53 can be stabilized by PTEN and promote its transcription. PTEN promoter deficiency demonstrated a defect in stabilizing and binding p53, contributing to PTEN reduction and alleviating its suppressive function in sporadic cancers and Cowden syndrome patients [84]. Pin1, polycomb group protein EZH2, AKT activation, and RAC1^P29S^ negatively mediate the transcription and function of PTEN [85–88]. Previous studies have indicated that mutations in the PTEN promoter are dispensable for common cancers. However, the potential role of the PTEN promoter in cancers needs more in-depth investigation.

##### The effect of deletion of other genes besides PTEN in the hyperactivity of PI3K/AKT/mTOR pathway

Besides PTEN deletion alone, deletion of both Lkb1 and Pten genes and mTOR excessive activation result in the development of ovarian carcinoma [89]. PTEN inactivation by S-nitrosylation could also induced PI3K/AKT Activation. PARK2 depletion mediated AMPK activity and promoted oxygen reaction leading to PTEN S-nitrosylation [90]. Inositol polyphosphate 4-phosphatase type II (INPP4B) acts as a negative regulator in PI3K activity, which promotes PIP2 to generate PIP3. INPP4B mutation led to the silence of PI3K signaling [91]. In mice kidney neoplasms, TSC2 exon 3 deletion contributed to the hyperactivity of mTOR, which resulted in Hes1 overexpression [92].

##### Hyperactivation of PI3K/AKT/mTOR signaling driven by gene fusion

Gene fusion is made by linking parts of two different genes during DNA from one chromosome moves to another chromosome, leading to fusion protein production. Fusion protein production has been found in colorectal cancer, myofibroma, B-lymphoblastic leukemia, glioblastoma, NSCLC, and thyroid carcinoma [93]. Gene fusions are commonly found in the PI3K/AKT/mTOR axis in cancer development. In the landscape of recurrent kinase fusions in solid tumors, the proportion of gene fusions was broadly different among various tumors, indicating diversity in the etiology of these neoplasms. PI3KCA and AKT fusions have been widely researched, and their functions are involved in PI3K/AKT/mTOR hyperactivation [94]. TBL1XR1–PIK3CA fusions were shown in invasive breast cancer and prostate cancer, which were driven by hormones [95]. Juxtaposing the promoter of the TBL1XR1 gene to the 5’ end of the intact PIK3CA coding sequence contributes to TBL1XR1–PIK3CA fusions, which causes an increase in PIK3CA mRNA [96]. LAMTOR1-Akt1 was observed in epithelioid cancer patients with tumorigenic driver function. In addition, BCAM-Akt2 and RPS6KC1–Akt3 were reported and validated in ovarian carcinoma and mammary cancer, respectively [97]. Hyperactivation and alterations of AKT proteins and their upstream and downstream effectors were generally researched in neoplasms of adults and pediatric malignancies, and only rare AKT fusions have been described [98–106].

In humans, the mTOR-TP53BP1 fusion gene is easily observed in colorectal carcinoma, mammary neoplasm, ovarian carcinoma, and lung carcinoma and regulates PI3K/AKT/mTOR signaling pathway activation. TFE3-mTOR, FKBP-mTOR, CHD1-mTOR, mTOR-CASZ1 and mTOR-TP53BP1 were found in cancers. However, their roles in tumorigenesis need further exploration [107, 108].

#### Transcriptional modifications drove PI3K/AKT/mTOR signaling pathway hyperactivation in cancer

In addition to genetic alterations leading to stable oncogenicity to promote oncogenesis and chemotherapy resistance, the production of proteins and expression of mRNA in PI3K/AKT/mTOR signaling pathways also participate in neoplasm growth [109]. Various effectors are involved in regulating the PI3K/AKT/mTOR pathway, such as promoter modification, microRNAs (miRNAs), and transcriptional control. MicroRNAs (miRNAs), small noncoding RNAs, regulate a third of the functional genomics at the posttranslational level. Upregulated Rictor expression was present in glioblastoma, advanced hepatocellular carcinoma (HC), SCLC, prostate carcinoma, cervical cancer, glioblastoma, mammary neoplasm, colorectal carcinoma, endometriosis, melanoma and esophageal squamous cell carcinoma (ESCC) [110, 111].

Distinct factors facilitate the signals in the pathways to regulate gene expression Promoter methylation could decrease the transcription of a targeted gene. PTEN promoter methylation is one of the most frequent sites in the PI3K/AKT/mTOR signaling pathway. TSC2 and TSC1 promoter methylation was significantly increased in breast carcinoma, oral squamous cell carcinoma, and tuberous sclerosis complex [112, 113]. The data show for the first time that methylation of the TSC2 promoter might cause a complete loss of tuberin in TSC2 cells and that the pathogenesis of angiomyolipomas might also originate from epigenetic defects in smooth muscle cells. TSC2 promoter methylation promoted the abnormal proliferation of smooth muscle-like cells, leading to TSC [114]. Both PI3K/AKT/mTOR and RAF/MEK/ERK can be activated by TBX1 suppression in thyroid adenocarcinoma, which is induced by its promoter methylation [115].

In human NSCLC cells, a decrease in miR-192-5p induces tumor development. Downregulation of miR-143, miR-145, and miR-101 in Burkitt’s lymphoma promotes tumor cell growth [116]. MiR-19a and miR-96 reduction suppress tumor cell proliferation through the PI3K/AKT pathway in HC cells [117]. MiR-425/489 is a target gene for the long noncoding RNA MHENC [118]. MiR-425/489 is increased in melanocytes. Knocking down MHENCR significantly inhibits melanocyte growth and causes cell cycle arrest and cell apoptosis.

Yang et al. reported that miR-1297 bound to the 3’ end of Meg3 and that miR-1297 could also bind to PTEN in testicular germ cell neoplasms [119]. The effect of miR-1297 on the 3’ end of PTEN mRNA expression could be suppressed by PTEN expression, leading to deactivation of AKT and reduction of cell proliferation. PTEN mRNA polyadenylation is frequently observed to regulate miRNA-mediated PTEN [120]. Polyadenylation of PTEN mRNAs is a dynamic process and results in different isoforms with distinct 3′UTRs [121]. Considering the lack of specificity of transcription factors in controlling PI3K/AKT/mTOR signaling expression, we did not discuss transcription factors and transcription modification in this review.

## Inhibitors of the PI3K/AKT/mTOR pathway

This pathway is one of the most common dysregulated pathways in tumors and a key signal in regulating tumor cell proliferation and apoptosis [122, 123]. The molecules in this pathway have drawn comprehensive attention in recent years. Many agents targeting components in this signaling pathway have been researched and assessed in animals and humans.

### PI3K inhibitors

**Fig. 2:**
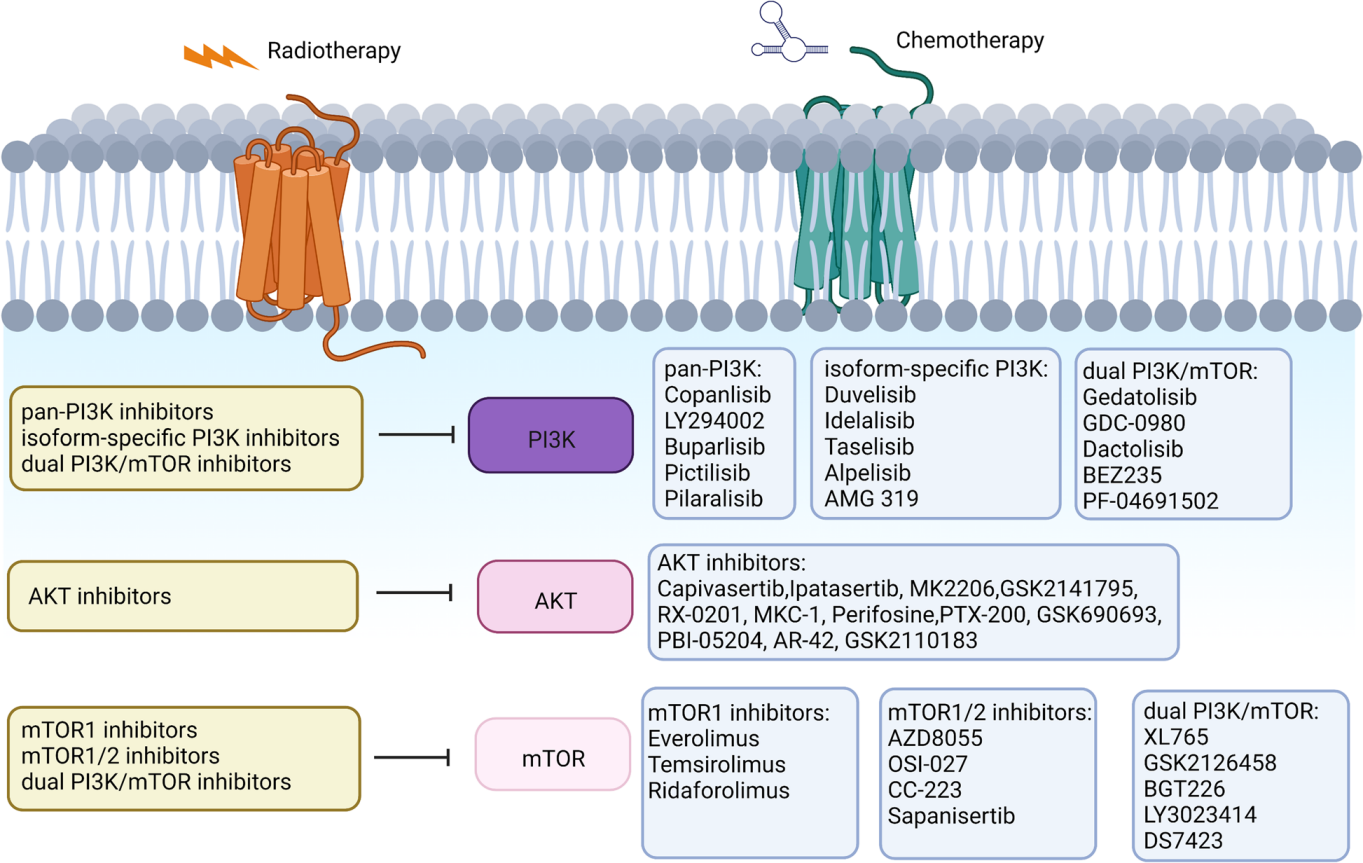
Inhibitors of the PI3K/AKT/mTOR pathway. Various classes of agents target different effectors of the PI3K/AKT/mTOR pathway

PI3K inhibitors were the primary agents developed and investigated in the PI3K/AKT/mTOR signaling pathways (Fig. 2) [124]. Various PI3K inhibitors were in development and can be separated into three categories based on their pharmacokinetic effect and interaction with ATP: pan-PI3K inhibitors, isoform-specific PI3K inhibitors and dual PI3K/mTOR inhibitors. Clinical trials have presented advanced antitumor treatment effects (Table 2) [125]. Compared with isoform-specific inhibition, class IA pan-PI3K inhibitors have been more comprehensively researched [126]. The off- and on-target effects of blocking all isoforms caused by pan-Class I inhibitors limited their use; however, their antitumor role was not influenced [127].

**Table 2:** Inhibitors of PI3K/AKT/mTOR

Inhibitors/natural products	Target	Studies found in ClinicalTrials.gov	Sponsor (Company)	Conditions	Study stage
Duvelisib (Copiktra/IP1-145)	p110δ	51	John Kirkwood	CLL, SLL, Relapsed/Refractory FL, Advanced Unresectable Melanoma, Relapsed or Refractory Lymphoma, and Recurrent/Metastatic HNSCC.	Approved
Alpelisib (BYL719)	p110α	97	Novartis Pharmaceuticals	Pancreatic Cancer, Colorectal Cancer,Esophageal Squamous Cell Carcinoma, Lung Cancer, Gastric Cancer, Recurrent or Metastatic HNSCC, Relapsed and Refractory Multiple Myeloma, Oropharyngeal Cancer, Breast Neoplasms, and Ovarian Cancer	Approved
Idelalisib (CAL-101/GS-1101/Zydelig)	p110δ	73	Gilead Sciences	CLL, Indolent Non-Hodgkin’s Lymphomas, Metastatic Pancreatic Ductal Adenocarcinoma, and Metastasis/Recurrence NSCLC	Approved
Umbralisib (RP5264/TGR-1202)	PI3Kδ	24	Eli Lilly and Company	CLL, Relapsed or Refractory Classical Hodgkin Lymphoma,and Mantle Cell Lymphoma,	I, II, III
AMG319	p110δ	2	Amgen	HNSCC, and Lymphoid Malignancy	I
INK11179 (Serabelisib/TAK 117/ MLN1117)	p110α	11	Millennium Pharmaceuticals	NSCLC, Clear-cell Metastatic Renal Cell Carcinoma, Advanced and Metastatic Gastric or Gastroesophageal Adenocarcinoma, and Endometrial Neoplasms	I, II
ME-401 (PWT-143)	p110δ	7	Kyowa Kirin	Relapsed or Refractory Indolent B-cell Non-Hodgkin’s Lymphoma, FL, and Marginal Zone Lymphoma	I, II
AZD6482	p110β	3	AstraZeneca	/	Preclinical
GSK2636771	p110β	8	GlaxoSmithKline	Gastric Cancer, Advanced Refractory Solid Tumors, Lymphomas, and Multiple Myeloma	I, II
AZD8186	p110β	4	AstraZeneca	Advanced Solid Tumors With PTEN or PIK3CB Mutations That Are Metastatic or Cannot Be Removed by Surgery	I, II
IPI-549	p110γ	5	Infinity Pharmaceuticals	Advanced Urothelial Carcinoma, HNSCC, Triple-Negative Breast Cancer, Renal Cell Carcinoma,and Ovarian Cancer	I, II
GDC-0077	p110α	6	Hoffmann-La Roche	Breast Cancer	I, II
GDC-0032 (Taselisib)	p1100α, p110δ, and p110γ	10	Genentech	Breast Cancer, Recurrent Squamous Cell Lung Carcinoma, and Stage IV Squamous Cell Lung Carcinoma	I, II, III
KA2237	PI3Kβ, and p110γ	1	Karus Therapeutics Limited	B Cell Lymphoma	I
Tenalisib (RP6530)	p110δ, and p110γ	10	Rhizen Pharmaceuticals SA	T Cell Lymphoma, Relapsed/Refractory CLL, Non Hodgkin Lymphoma, Locally Advanced Breast Cancer, and Metastatic Breast Cancer	I, II
CUDC-907 (Fimepinostat)	PI3Kα and HDAC1/2/3/10	7	Curis	Relapsed or Refractory Solid Tumors, CNS Tumors, or Lymphoma, Relapsed and/or Refractory Diffuse Large B-cell Lymphoma Including With Myc Alterations, Metastatic and Locally Advanced Thyroid Cancer,and Prostate Cancer	I, II
Dactolisib (BEZ 235)	PI3K/mTOR	27	Novartis Pharmaceuticals	Renal Cancer, Castrate-resistant Prostate Cancer, Metastatic Breast Cancer, Pancreatic Neuroendocrine Tumors, Endometrial Cancer, Relapsed or Refractory Acute Leukemia, and Glioblastoma	I, II, III
GDC-0980 (Apitolisib)	PI3K/mTOR	12	Genentech	Renal Cell Carcinoma, Endometrial Carcinoma, Non-Hodgkin’s Lymphoma, Breast Cancer,and Prostate Cancer	I, II
SF1126	PI3K/mTOR	4	SignalRX Pharmaceuticals	Metastatic Squamous Neck Cancer With Occult Primary Squamous Cell Carcinoma, and Advanced Hepatocellular Carcinoma	I, II
XL765 (SAR245409)	PI3K/mTOR	11	Sanofi	Glioblastoma, NSCLC, Malignant Gliomas, Breast Cancer, Indolent Non-Hodgkin Lymphoma, Mantle Cell Lymphoma, CLL, and Ovarian Cancer	I, II
PF-04691502	PI3K/mTOR	6	Pfizer	Neoplasm Malignant	I
GSK2126458	PI3K/mTOR	3	GlaxoSmithKline	Solid Tumours	I
BGT226	PI3K/mTOR	2	Novartis	Advanced Breast Cancer	I, II
LY3023414	PI3K/mTOR	16	Eli Lilly and Company	Relapsed or Refractory Advanced Solid Tumors, Non-Hodgkin Lymphomas, Triple Negative Breast Cancer, Endometrial Cancer, Non-small Cell Lung Cancer Metastatic, Prostate Cancer Metastatic, and Pancreatic Ductal Adenocarcinoma	I, II
PF-05212384 (Gedatolisib/PKI-587)	PI3K/mTOR	19	Pfizer	AML, Breast Cancer, Endometrial Neoplasms, Metastatic Colorectal Carcinoma, NSCLC, Ovary Cancer, Endometrial Cancer, SCLC, HNSCC, and Pancreatic Cancer	I, II
DS7423	PI3K/mTOR	1	Daiichi Sankyo, Inc.	Colorectal Cancer, and Endometrial Cancer	I
GDC0084(Paxalisib)	PI3K/mTOR	8	Bayer	Glioblastoma, Diffuse Midline Gliomas, Primary CNS Lymphoma, Non-Hodgkin Lymphoma of Extranodal Site, and Breast Cancer	I, II
PWT33597	PI3K/mTOR	1	Pathway Therapeutics	Advanced malignancies	I
PQR309 (Bimiralisib)	PI3K/mTOR	9	PIQUR Therapeutics AG	HNSCC, Metastatic HER2 Negative and Triple-negative Breast Cancer, Relapsed or Refractory Primary CNS Lymphoma, and Relapsed or Refractory Lymphoma	I, II
P7170	PI3K/mTOR	1	Piramal Enterprises Limited	Advanced Refractory Solid Tumors	I
SF-1126	PI3K/mTOR	4	SignalRX Pharmaceuticals	Neuroblastoma, Advanced Hepatocellular Carcinoma, and Metastatic Squamous Neck Cancer With Occult Primary Squamous Cell Carcinoma	I, II
Rigosertib (ON-01910)	pan-class I PI3K	38	Onconova Therapeutics	Myelodysplastic Syndrome, Chronic Myelomonocytic Leukemia, Refractory Leukemia, Squamous Cell Carcinoma, Ovarian Cancer, and Metastatic Pancreatic Adenocarcinoma	I, II
CH5132799	pan-class I PI3K	1	Chugai Pharma Europe	Solid Tumors	I
Copanlisib (BAY 80 − 6946)	pan-class I PI3K	70	Bayer	Lymphomas, or Multiple Myeloma, Radioiodine-Refractory Thyroid Cancers, HNSCC	Approved
LY294002	pan-class I PI3K	1	SignalRX Pharmaceuticals	Neuroblastoma	I
Buparlisib (BKM120/LDE225)	pan-class I PI3K	90	Novartis Pharmaceuticals	NSCLC, Prostate Cancer, Lymphoma, HNSCC, Metastatic Colorectal Cancer, Metastatic Pancreatic Cancer, Breast Cancer, Metastatic Transitional Cell Carcinoma of the Urothelium, Renal Cell Carcinoma, Relapsed or Refractory CLL, Glioblastoma, Treatment for Metastatic or Locally Advanced Cervical Cancer, Esophageal Cancer, Thyroid Cancers, Advanced Endometrial Cancer, and BRAF Mutant Metastatic Melanoma	I, II, III
Pictilisib (GDC 0941)	pan-class I PI3K	17	Genentech	NSCLC, Breast Cancer, Non-Hodgkin’s Lymphoma, and Glioblastoma	I, II
Pilaralisib (SAR 245,408/XL 147)	pan-class I PI3K	17	Sanofi	Endometrial Cancer, Breast Cancer, NSCLC, Ovarian Carcinoma, Glioblastoma, and Astrocytoma	I, II
SF1126	pan and dual first-in-class PI3K/BRD4	4	SignalRX Pharmaceuticals	Advanced Hepatocellular Carcinoma, Neuroblastoma, and Metastatic Squamous Neck Cancer,	I, II
PX866	pan-class I PI3K(from Wortmannin)	7	Oncothyreon Canada	Prostate Cancer, Glioblastoma, Advanced BRAF-mutant Cancers, Incurable Metastatic Colorectal Carcinoma Incurable Progressive, NSCLC, and HNSCC	I, II
Capivasertib (AZD-5363)	Akt1 Akt2 Akt3	22	Parexel	Relapsed or Refractory B-cell Non-Hodgkin Lymphoma, Triple Negative Breast Neoplasms, Hormone-Sensitive Prostate Cancer, Recurrent or Refractory Endometrial Cancer, and Meningioma, Lymphoma	Approved
Ipatasertib (GDC- 0068	Akt1 Akt2 Akt3	26	Genentech	Ovarian Neoplasms, Breast Cancer, Gastric Cancer, Prostate Cancer, Glioblastoma, Recurrent or Metastatic HNSCC, Epithelial Ovarian Cancer, and Endometrial Cancer	Approved
MK2206	Akt1 Akt2 Akt3	32	AstraZeneca	Breast Cancer, Prostate Cancer, Lymphoma, and Advanced Gastric Cancer	Approved
GSK2141795	Akt1 Akt2 Akt3	12	Novartis	Cervical Cancer, Melanoma, Acute Myeloid Leukemia, Breast cancer,Relapsed or Refractory Multiple Myeloma, Uveal Melanoma, and Endometrial Adenocarcinoma	I, II, III
Ipatasertib	Akt1 Akt2 Akt3	52	Genentech	Metastatic Breast Cancer, Head and Neck Cancer, Castrate Resistant Prostate Cancer, Ovarian Cancer, Metastatic Prostate Cancer,Gastric Cancer, Endometrial Cancer, and NSCLC	I
RX-0201	Akt1 Akt2 Akt3	2	Rexahn Pharmaceuticals	Metastatic Pancreatic Cancer, and Metastatic Renal Cell Cancer	I, II
MKC-1	Akt1 Akt2 Akt3	9	CASI Pharmaceuticals	Pancreatic Cancer, Refractory Hematologic Malignancies, Ovarian Cancer, Endometrial Cancer, Breast Cancer, NSCLC,and Colorectal Cancer	I, II
Perifosine	Akt1 Akt2 Akt3	44	SCRI Development Innovations	Colorectal Cancer, CLL, SLL, Gastrointestinal Stromal Tumors, Recurrent/Progressive Malignant Gliomas, Renal Cell Carcinoma, Multiple Myeloma, Kidney Cancer, Recurrent Prostate Cancer, Head and Neck Cancer, Ovarian Cancer, Pancreatic Cancer, Breast Cancer, Melanoma, and Endometrial Cancer	Preclinical
PTX-200 (Triciribine)	Akt1 Akt2 Akt3	3	Prescient Therapeutics	Acute Leukemia, Ovarian Cancer, and Breast Cancer	I, II
GSK690693	Akt1 Akt2 Akt3	2	GlaxoSmithKline	Relapsed or Refractory Hematologic Malignancies	I, II
PBI-05204	Akt1 Akt2 Akt3	2	Phoenix Biotechnology	Pancreatic Cancer	I, II
AR-42 (OSU-HDAC42/NSC-D736012/REC-2282)	Akt1 Akt2 Akt3	6	Recursion Pharmaceuticals	Renal Cell Carcinoma, Soft Tissue Sarcoma, Recurrent Plasma Cell Myeloma, Advanced or Relapsed Multiple Myeloma, CLL, and Lymphoma	I, II
GSK2110183	Akt1 Akt2 Akt3	10	GlaxoSmithKline	Refractory Multiple Myeloma, CLL, Recurrent Platinum-resistant Ovarian Cancer, and Hematologic Malignancies	I, II, III
SR13668	Akt1 Akt2 Akt3	1		Healthy Volunteers	I
AZD5363	Akt1 Akt2 Akt3	32	AstraZeneca	Breast Cancer, Prostate Cancer,Lymphoma, Advanced Gastric Cancer, Endometrial Adenocarcinoma, Recurrent Ovarian Carcinoma, Recurrent Uterine Corpus Carcinoma, Meningioma, and Metastatic NSCLC	I, II
Everolimus (RAD001)	mTORC1	1152	Pfizer	Solid Tumor	I, II, III
Temsirolimus (CCI-779/Rapamycin/sirolimus)	mTORC1	1060	Millennium Pharmaceuticals	Solid Tumor	I, II, III
Ridaforolimus (MK-8669/Deforolimus/ AP23573)	mTORC1	46	Ariad Pharmaceuticals	NSCLC, Soft Tissue Sarcoma, Breast Neoplasms, Prostate Cancer, Endometrial Cancer, Ovarian Cancer, and Relapsed or Refractory Hematologic Malignancies,	I, II
AZD2014	mTORC1/2	32	AstraZeneca	Stomach Neoplasms, Prostate Cancer, Relapsed or Refractory Diffuse Large B-Cell Lymphoma, Meningioma, Triple-Negative Breast Cancer, Squamous Cell Lung Cancer, Non-squamous Cell Lung Cancer With KRAS Mutations, Non-squamous Cell Lung Cancer With Wild-type KRAS, Metastatic Clear Cell Renal Carcinoma, SCLC, Muscle Invasive Bladder Cancer	I, II
AZD8055	mTORC1/2	5	AstraZeneca	Recurrent Gliomas, and Advanced Hepatocellular Carcinoma,	I
OSI-027	mTORC1/2	1	Astellas Pharma	Any Solid Tumor or Lymphoma	I
CC-223	mTORC1/2	6	Celgene	Advanced Solid Tumors, Non-Hodgkin Lymphoma or Multiple Myeloma, NSCLC, and Hepatocellular Carcinoma	I, II
Sapanisertib (MLN0128/TAK-228 / INK128)	mTORC1/2	44	Millennium Pharmaceuticals	Metastatic Breast Cancer, Soft Tissue Sarcoma, Thyroid Cancer, Hepatocellular Carcinoma, Metastatic Castration-Resistant Prostate Cancer, Merkel Cell Carcinoma, Clear-cell Metastatic Renal Cell Carcinoma, Relapsed or Refractory Multiple Myeloma, Recurrent Glioblastoma, Lymphoma, Urothelial Carcinoma, Women With Endometrial Cancer, Metastatic or Refractory Pancreatic Neuroendocrine Tumor, Stage IV or Recurrent Lung Cancer, and Locally Advanced or Metastatic Bladder Cancer	I, II

*CLL * Chronic lymphocytic leukemia, *SLL* Small Lymphocytic Lymphoma, *FL* Follicular Lymphoma, *HNSCC* Head and Neck Squamous Cell Carcinoma, *NSCLC*, Non Small Cell Lung Cancer, *CNS * Central Nervous System, *AML *Acute Myeloid Leukemia, *SCLC* Small Cell Lung Cancer

#### Pan-PI3K inhibitors

Buparlisib (BKM120) is an ATP-competitive pan class l PI3K inhibitor that can also affect mTOR and Vps34 at higher doses [128]. Buparlisib has already been assessed in a phase 3 trial [129]. Buparlisib exhibited superior antitumor effects in human cells in vitro [130, 131]. In vivo, buparlisib presents excellent oral bioavailability and superior antitumor activity in mouse models. In a dose-escalation study of buparlisib, the most frequent buparlisib-related adverse effects (AEs) included rash, hyperglycemia, diarrhea, anorexia, mood alteration, decreased appetite, nausea and abnormal hepatic function [132]. In a phase I trial, the AEs increased in patients treated with buparlisib (40 mg daily) combined with standard mFOLFOX6 compared with either buparlisib or mFOLFOX6 [133]. In patients treated bevacizumab with BKM120, no encouraging efficacy was observed in glioblastoma tumors when bevacizumab was combined with BKM120 [134].

Based on the safety dose in solid tumors treated with chemotherapy combined with BKM120, the combination of BKM120 at a safe dose and radiotherapy was applied to advanced non-small cell lung carcinoma [135]. This therapeutic regimen was well tolerated, and hypoxia could be improved by PI3K inhibition, which indicated that BKM120 may act as a radiosensitizer. The maximum tolerated dose of BKM120 was 100 mg/day in solid tumors; however, the maximum tolerated dose of BKM120 was 80 mg/day in leukemias. Leukemia patients treated with the maximum tolerated dose demonstrated modest efficacy. BKM120 monotherapy demonstrated promising efficacy and manageable AEs in advanced ESCC patients in a phase II study [136]. However, buparlisib monotherapy was connected to an unfavorable safety profile and unsatisfactory anticancer activity in advanced or recurrent endometrial carcinoma [137]. The study was withdrawn before recruitment was finished because of AEs. In the BELLE-3 trial, the combination of buparlisib with fulvestrant was not recommended in postmenopausal, hormone receptor (HR)-positive, HER2-negative, advanced breast cancer for the safety profile [138]. The use of buparlisib monotherapy in recurrent glioblastoma was limited by efficacy. Patients treated with buparlisib in combination with paclitaxel had longer survival times in relapsed or metastatic head and neck squamous cell carcinoma patients [139]. Then, buparlisib plus paclitaxel was recommended as a second-line therapy.

Copanlisib (BAY 80 − 6946) is a panclass I PI3K inhibitor that can inhibit all four PI3K class-I isoform activations [140]. The maximum tolerated dose of copanlisib monotherapy in patients with advanced solid tumors and non-Hodgkin lymphoma (NHL) was determined in the NCT00962611 clinical trial [141]. Copanlisib has promising antitumor activity in these patients, particularly in NHL patients. The most common AEs related to copanlisib included hyperglycemia, nausea, and hypertension. Grade ≥ 3 AEs related to copanlisib were hyperglycemia, hypertension, and rash. Another study developed on advanced or refractory solid tumors, and copanlisib was well tolerated in Japanese patients [142]. The most common toxicities in Japanese patients were hyperglycemia and hypertension. The maximum tolerated dose of copanlisib in Chinese patients with relapsed or refractory NHLs was at a lower dose and presented promising effects [143]. The COUP-1 trial assessed the effectiveness and AEs of copanlisib in combination with rituximab in marginal zone lymphoma patients, which demonstrated the potential for the clinical and translational use of copanlisib [144]. In a phase II study of copanlisib in various lymphomas, the results showed that intravenous copanlisib could offer promising efficacy and manageable toxicity [145]. Further studies on copanlisib in peripheral T-cell and mantle cell lymphomas were carried out. In PIK3CA mutation patients, the over response rate was 16% with tolerable AEs, including hyperglycemia (76%), fatigue (48%), diarrhea (44%), hypertension (40%), and nausea (40%). The long-term efficacy and toxicity of copanlisib in patients with relapsed or refractory indolent lymphoma exhibited progression-free survival, and overall survival was 12.5 months and 42.6 months, respectively. Approximately twenty-six patients received copanlisib four over one year [146]. In phase III of Copanlisib plus rituximab in indolent NHL, Copanlisib plus rituximab could improve progression-free survival compared with placebo plus rituximab [147]. Overall, serious AEs were largely unchanged and tolerable, with no new cases or grade 5 events in the undergoning and completed clinical trials. Treatment-emergent AEs caused by copanlisib did not contribute to increased incidence or worsening prognosis. The results in the study demonstrated that intravenous copanlisib led to a sustained, intensive treatment response without increasing treatment-emergent AEs, similar to other orally administered PI3K inhibitors [148].

In addition to buparlisib (BKM120) and copanlisib (BAY 80 − 6946), LY294002, pictilisib (GDC 0941), pilaralisib (SAR 245,408 and XL 147), SF1126, ZSTK474, rigosertib (ON-01910) and CH5132799 are pan class l PI3K inhibitors with ongoing clinical studies [149]. Copanlisib (BAY 80 − 6946) is the only pan-PI3K inhibitor approved for tumor therapy and is a potential treatment for malignat solid tumors and hematologic malignancies [141]. In 2017, the FDA administered copanlisib to patients with recurrent follicular lymphoma who were treated with two or more previous systemic therapies based on the outcomes of the CHRONOS-1 trial [146].

#### Isoform-selective PI3K inhibitors

Compared with pan-PI3K inhibitors, isoform-selective PI3K inhibitors target one of the isoforms in PI3K, which decreases AEs and enhances targets [150]. Patients treated with isoform-selective PI3K may be selected and identified with sensitivity and resistance markers [151]. Preclinical trials of isoform-specific PI3K inhibitors in cancer are limited. GS-1101 (idelalisib/CAL-101), IPI145 (duvelisib, INK-1197) and alpelisib (BYL719) have been approved by the FDA for tumor therapy. In 2014, idelalisib was approved for treating chronic lymphocytic leukemia (CLL), relapsed follicular B-cell NHL, and relapsed small lymphocytic lymphoma (SLL) [152]. In 2018, duvelisib was approved in relapsed or refractory CLL or SLL after more than two previous therapies [153]. In 2019, alpelisib (a PI3KCA inhibitor) was approved for treating HR-positive, EGFR-negative, PI3K mutation, advanced or metastatic mammary neoplasms [154]. Based on the outcomes of the CBYL719 × 2101 trial, alpelisib showed promising results and tolerable toxicity in PIK3CA-mutant tumor patients, demonstrating that isoform-selective PI3K inhibitors combined with other antitumor regimens may be efficient in treating PIK3CA-mutant tumors [155]. The newest PI3K inhibitor approved by the FDA was umbralisib in 2021, which is efficient in treating lymphoma according to the UTX-TGR-205 trial [156].

#### Dual PI3K/mTOR inhibitors

Considering that both PI3K and mTOR are members of the same PIKK super family of kinases accompany with similar structural isoforms and responses, inhibitors inhibiting both PI3K and mTOR targets were found through research on mTOR inhibitors [157]. The administration of BEZ235 to improve glucocorticoid resistance in pediatric T-ALL and the use of PKI-587 to decrease cancer cell proliferation in T-ALL demonstrated that dual PI3K/mTOR inhibitors significantly improved the treatment effect compared with inhibiting mTOR or PI3K alone [158–160].

In a phase Ib dose-finding study of BEZ235, diarrhea (58%), mucositis (58%), and nausea (42%) were the most frequent toxicities of all grades. Mucositis was the most common grade 3 AE [161]. No grade 4 BEZ235-related toxicity was observed, and no drug-related hospitalizations or deaths were found. BEZ235 monotherapy at 300 mg BID orally was recommended in a phase II study, which is well tolerated, and no objective responses were found [162]. Further studies of BEZ235 have been developed in monotherapy or combination with other agents in various solid tumors and hematologic malignancies [163]. Although the outcomes of these studies showed that BEZ235 is generally tolerated, the clinical response is restricted [164]. There are no new clinical trials of BEZ235 developed on solid tumors. Some acute lymphoblastic leukemia patients may have some alterations in the PI3K/AKT/mTOR signaling pathway, and BEZ235 had promising effects in this small subset of patients [165]. AML did not benefit from BEZ235 treatment. In metastatic renal cell carcinoma (RCC), apitolisib (GDC-0980) was less effective than everolimus [166]. Increased toxicity was observed in the trials.

Voxtalisib (SAR245409, XL765) is a potent inhibitor targeting class-I PI3Ks, mTORC1 and mTORC2 [167]. In vitro, voxtalisib inhibited the phosphorylation of PI3K and controlled mTOR effector incorporation in malignant tumor cells [168]. In a phase Ib study of voxtalisib in patients with advanced malignant tumors, pimasertib (90 mg) plus voxtalisib (70 mg) demonstrated poor long-term tolerability and no faverable survival in advanced solid tumor patients [169]. Diarrhea (75%), fatigue (57%), and nausea (50%) were frequently observed in the pimasertib plus voxtalisib group. In relapsed or refractory NHL or CLL, voxtalisib was tolerable when it was given at 50 mg orally twice a day [170]. Voxtalisib (40 mg b.i.d.) plus temozolomide with or without radiotherapy presented a favorable safety profile in patients with high-grade gliomas. Among these patients, 4% had a partial response, and 68% had stable disease.

Compared with voxtalisib and BEZ235, PQR309 (bimiralisib) showed a better ability to transfer the brain blood barrier (BBB) [171]. PQR309 monotherapy or in combination with other small molecular inhibitors demonstrated promising antitumor activity in lymphomas [172]. In a phase I dose-escalation study, dose-limiting toxicities were not exhibited in advanced solid lymphoma patients. Seventy precention patients were found to have grade 3 treatment emergent AEs, and approximately 30% of patients had grade 4 treatment emergent AEs. 28% of patients discontinued treatment. PQR530 and PQR620 also had a better ability to transfer the BBB [173].

GDC-0084 is another oral and brain-penetrant dual PI3K/mTOR inhibitor. The usual PI3K/mTOR-related AEs were observed after GDC-0084 treatment. The first phase I study of GDC-0084 in patients with progressive or recurrent glioma identified that GDC-0084 could cross the blood‒brain barrier [174]. The results indicated that GDC-0084 is a potential compound in brain metastatic mammary neoplasms with a dysregulativity of PI3K/mTOR signals conferred by PIK3CA mutations.

Many dual inhibitors have been developed and applied in xenograft mouse models and malignant tumor cell lines with promising effects on tumors. PKI-587 improved the radiosensitivity and oxaliplatin sensitivity of HC via the PI3K/AKT/mTOR pathways to reduce DNA damage repair [175]. The application of PKI-587 led to broad spectrum cancer cell stasis and cell apoptosis. PIK3CA mutations caused by activation of the WNT/β-catenin signaling pathway may decrease colorectal cancer cell sensitivity to the dual PI3K/mTOR inhibitor PKI-587 [176]. PKI-587 plus cofetuzumab pelidotin in metastatic triple-negative breast cancer (TNBC) showed moderate toxicity and promising clinical activity [177]. PKI-587 combined with carboplatin and paclitaxel in clear cell ovarian cancer exhibited similar trends [178]. The administration of PKI-587 is well tolerated by weekly intravenous infusion rather than daily oral use in recurrent endometrial cancer [179]. The clinical response in the PKI-587/stathmin-low arm was slightly satisfactory, while that of the gedatolisib/stathmin-high arm did not meet the clinical benefit response criteria.

In addition to the mentioned small molecule inhibitors, dual PI3K/mTOR inhibitors such as GSK2126458, SF1126, LY294002, PF-04691502, LY302341, and PWT33597 have shown favorable antitumor efficacy in various malignant neoplasms [180]. In a cellular assay, GSK2126458, an oral inhibitor, inhibited the growth and aggression of pancreatic cancer cells [181]. SF1126, an Arg-Gly-Asp (RGD)-conjugated LY294002 prodrug, can inhibit both the PI3K-Akt-mTOR and BRD4 cascades. In colorectal cell lines and primary human colon cancer cells from human tumors, SF1126 inhibited cell growth and apoptosis and blocked the cell cycle [182]. Administration of SF1126 led to tumor angiogenesis in tumor tissues. Combining different chemotherapeutic agents with PF-04691502 promoted breast cancer cell apoptosis [183]. PF-04691502 could also promote radiosensitivity of gastroenteropancreatic neuroendocrine tumors. BGT226, an imidazoquinoline derivative, is an ATP-competitive dual PI3K/mTORC1/C2 inhibitor [184]. LY302341 presented promising antitumor activity in esophageal adenocarcinoma [185]. Clinical research on PWT33597 in advanced malignancies has been developed, but no results have been posted.

### AKT inhibitors

AKT is a kind of effector in the PI3K/AKT/mTOR pathway of tumors and is a potential target in treating cancer [186]. The AKT kinase family includes three isoforms, Akt1, Akt2, and Akt3 (Table 2).

Phosphorylation and dephosphorylation of AKT regulate the activation of Akt-dependent behavior [187]. ATP-competitive inhibitors promote dephosphorylation of AKT activity by inhibiting ATP activity [188]. Allosteric inhibitors interact with the AKT substrate by inducing conformational transitions of their enzymatic structure [189]. Irreversible inhibitors are uncommon AKT inhibitors. Both ATP-competitive inhibitors and allosteric inhibitors present potential effects in cancer cells [190]. ATP-competitive inhibitors contributed to AKT activation via inhibition of the pH and exposure of the ATP-binding pocket. Allosteric inhibitors block AKT on the plasmalemma and inhibit AKT activity [191].

In the first human study of capivasertib, capivasertib monotherapy showed clinical significance in Akt1 E17K-mutant metastatic breast cancer patients with estrogen receptor (ER) positivity [192]. Gastrointestinal events (diarrhea, vomiting, nausea) were the most frequent AEs related to capivasertib treatment in the D3610C00001 trial [193]. Approximately sixty patients experienced grade ≥ 3 AEs, including hyperglycemia, diarrhea, and maculopapular rash. The combination of capivasertib and enzalutamide is tolerable in metastatic castration-resistant prostate cancer and has a favorable prognosis. In the PAKT trial, capivasertib plus first-line paclitaxel therapy for metastatic TNBC led to extensively better progression-free survival and overall survival, which identified the role of capivasertib in TNBC treatment [194]. According to the FAKTION trial, the median progression-free survival of metastatic, ER-positive breast cancer patients treated with capivasertib plus fulvestrant was 10.3 months compared with 4.8 months in patients treated with fulvestrant plus placebo [195].

Ipatasertib (GDC-0068), which targets AKT signaling, showed acceptable AEs and a favorable prognosis in AKT-activated tumors [196]. No difference was found between the survival of ipatasertib/mFOLFOX6 and placebo/mFOLFOX6 (NCT01896531). In the IPATential150 trial, ipatasertib plus abiraterone prolonged the survival of metastatic prostate cancer patients with PTEN loss compared with standard regimens plus placebo (approximately 19 months vs. approximately 17 months) [197]. 70% of patients administered ipatasertib plus abiraterone had grade ≥ 3 AEs. Toxicity related to placebo plus abiraterone resulted in discontinuation in 5% (28/546) of prostate cancer patients. However, 21% (116/551) of patients in this trial were treated with ipatasertib plus abiraterone because of AEs. Ipatasertib plus paclitaxel prolonged survival compared with paclitaxel monotherapy (6.2 months vs. 4.9 months). In cohort B of the IPATunity130 randomized phase 3 trial, adding ipatasertib to paclitaxel did not have a promising effect in PI3K pathway-mutant HR-positive unresectable locally advanced/metastatic malignant mammary tumor patients. Referencing AEs, the administration of ipatasertib decreased the proportion of diarrhea, neutrophil count decrease, neutropenia, peripheral neuropathy, and peripheral sensory neuropathy [198].

SPY 2 is a phase II study elevating the response of MK-2206 in combination with standard taxane- and anthracycline-based neoadjuvant therapy [199]. In the I-SPY 2 trial, MK-2206 contributed to higher complete response rates in HR-negative and HER2-positive breast cancer [200]. The median progression-free survival and overall survival for recurrent endometrial cancer patients treated with MK-2206 were 2.0 months and 8.4 months, respectively. Rash, fatigue, nausea, hyperglycemia, diarrhea, fever, and vomiting were the most common AEs related to MK-2206. Compared with standard chemotherapy regimens, the activity of MK-2206 in recurrent endometrial cancer was limited [201]. Administration of MK-2206 demonstrated amazing effectiveness in uterine serous cancer.

### mTOR inhibitors

The mechanistic target of rapamycin (mTOR) is a ser/thr kinase that belongs to the PIKK family [202]. MTOR contains two multiprotein complexes called mTORC1 and mTORC2 [203]. MTORC1 participates in multiple growth factor signals to promote cell growth, whereas mTORC2 is primarily responsible for cell proliferation and survival. Rapamycin is a widely known mTOR inhibitor that has been developed in various solid tumors and hematologic malignancies [204]. Rapamycin was primarily separated from the soil on Rapanui Island and could be used as an anti-fungal agent [205]. Rapamycin integrates antitumor cell growth and acts as an immune suppressant. Rapamycin directly combines with FKBP12 targets, which immediate rapamycin to regulate mTORC1 activity [206]. Two allosteric mTORC1 inhibitors, temsirolimus (CCI-779) and everolimus (RAD001), have been approved for the treatment of cancer [207]. Both temsirolimus and everolimus are derivatives from rapamycin [208]. The activity mechanisms of temsirolimus and everolimus were similar to that of rapamycin, which binds to FKBP12 to encourage mTOR activity [209]. Temsirolimus and everolimus disturbed Raptor binding to mTOR, contributing to mTORC1 isoform decomposition and mTORC1 inactivation [210].

A phase I trial of temsirolimus demonstrated a higher overall response rate (ORR) of advanced RCC [211]. A phase II trial exhibited promising effects in patients with metastatic renal cell carcinoma [212]. A phase III trial of temsirolimus plus other chemotherapies promoted the survival of metastatic RCC patients [213]. Overall, temsirolimus is a potent agent in treating RCC [214]. Temsirolimus plus sorafenib in advanced HC demonstrated acceptable toxicity. No grade 5 events were observed. The most frequent drug-related grade ≥ 3 AEs were hypophosphatemia, thrombocytopenia, and rash in this phase II trial. The median time to progression of advanced HC patients who received temsirolimus plus sorafenib was 3.7 months, with 14% of patients reaching a time to progression of at least 6 months [213]. The median overall survival was 8.8 months. Everolimus is another well-researched derivative from rapamycin, including advanced RCC, HR-positive/HER2-negative mammary neoplasms, pancreatic neuroendocrine tumors, and ependy malignant cell astrocytoma [215]. Everolimus monotherapy plus capecitabine and oxaliplatin is presently being evaluated in phase I and phase II clinical trials of patients with gastric carcinoma [216]. The first-generation mTOR inhibitors temsirolimus and everolimus can inhibit mTORC1, resulting in activation of the MAPK signaling pathway via PI3K. Second-generation mTOR inhibitors inhibited both TORC1 and mTORC2 [217]. AZD8055 is a second-generation mTOR inhibitor that inhibits protein synthesis in HCs, inhibits malignant mammary tumor cell proliferation and reduces tamoxifen resistance [218].

AZD2014 is another second-generation mTOR inhibitor that attenuates myeloid-derived suppressor cell recruitment and blocks cell proliferation in ovarian cancer [219]. AZD2014 increased docetaxel sensitivity and overcame docetaxel resistance in prostate carcinoma cells [220]. AZD2014 in tumors has been developed in phase I and II clinical trials. In a phase I/II randomized clinical trial, AZD2014 plus anastrozole showed therapeutic benefit and had manageable toxicity in women with HR-positive recurrent or metastatic carcinoma of the endometrium [221]. In the AcSé-ESMART trial, the absence of clinical response and deficient target management in the AZD2014-treated adult subgroup led to the termination of the study [222]. However, a phase II study claimed that dual mTORC1 and mTORC2 inhibitors were not superior to mTORC1 inhibitors in relapsed or refractory diffuse large B-cell lymphoma [223]. Second-generation mTOR inhibitors did not confer advantages over second-generation mTOR inhibitors. Approximately 30% treated with mTORC1 inhibitors had a clinical response. In diffuse large B-cell lymphoma, 6% of patients achieved partial response, with no complete response [224]. 20% of patients reached stable disease after six cycles of treatment. The effect of AZD2014 in metastatic clear cell RCC is unclear. Currently, several dual mTORC1/2 inhibitors are undergoing clinical tests for adult and pediatric tumor therapy, such as OSI-027, CC-223, and MLN0128 [225].

## Overview of the RAF/MEK/ERK signaling pathway and its deregulation in cancer

### Function of the RAF/MEK/ERK signaling pathway

RAF/MEK/ERK transfer signals from receptors on the cytomembrane to regulate transcription, which promotes protein synthesis and other functions (Fig. 1) [226]. This pathway has been comprehensively researched because of its role in regulating cell apoptosis, which makes inhibitors targeting the components in this pathway have potential antitumor effects. The RAF/MEK/ERK pathway, also known as the MAPK pathway, transmits signals from receptors on the cell surface to the nucleus to promote transcription [227]. This pathway could be activated in various cancers by overexpression of B7-H3, upregulated expression of decidual protein induced by progesterone, overexpression of HOXA3, and aberrant expression of the COX2/PGE axis [228]. This signaling pathway also has enormous effects on promoting tumor cell apoptosis by DR5 expression, ALDH6A1 decrease, and KIF15 expression [226]. The pathway participates in tumor cell growth, cell cycle block, apoptosis, cell adhesion and differentiation.

### Construction of the RAF/MEK/ERK signaling pathway

#### The role of RAS in regulating the RAF/MEK/ERK signaling pathway

The complexity of this pathway was gradually observed with the academic paper published, including many factors in the transcription factor, apoptosis-promoting, and caspase executioner families [229]. Transcription of RAF genes could improve the phosphorylation of downstream proteins via MEK and ERK to control cancer cell apoptosis. The pathway positively or negatively regulates cancer cell apoptosis via different signals [230]. Alternations at upstream receptors, RAS, B-Raf and other genes contribute to abnormal RAF activation, which results in unnatural signaling pathway activity [231]. RAS, the key regulator and upstream protein of the RAF/MEK/ERK pathway, consists of four small GTPases [232]. RAS activation induced by epidermal growth factor promoted GTPase binding with upstream receptor tyrosine kinase signaling. The RAS gene family includes N-Ras, H-Ras, and K-Ras, which are the most commonly activated in human neoplasms. RAS alterations have been reported to be associated with poor overall survival. S-phase could be induced by V-Raf, indicating that RAF could be regulated by RAS or in parallel with RAS [233]. It was also confirmed by studies of Drosophila and C. elegans that RAF is understream of RTKs and RAS [234].

#### The cascade signaling in the RAF/MEK/ERK signaling pathway

RAF included three isoforms: A, B and C. The supporting evidence for C-Raf serving as a cellular oncogene was not sufficient. After stimulation of receptors on the cell surface for RAF activation, C-Raf was phosphorylated at the S43, S259 and S621 sites, which maintained kinase activity. A-Raf is rarely found to be mutated in neoplasms and is one of the weakest effectors in promoting MEK1. B-Raf is the strongest isoform that promotes MEK activity [235–242]. ERK is a downstream gene of a stable module stimulated by RAF ser/thr kinases. RAF activates ERK1/2 by stimulating MAPK/ERK kinase (MEK)1/2 dual-specificity protein kinases [243].

Collectively, the signaling pathway is a critical pathway in tumors and could act as a promising approach in therapy. Inhibitors in the pathway have been developed and are undergoing clinical trials [244].

## Mutations in the RAF/MEK/ERK Signaling Cascade

Gene alterations in the signaling cascade can be divided into two categories: driver of neoplasms (RAF mutations) or indicator of worse survival (MEK and ERK mutations) [245]. In this section, mutations in the RAF/MEK/ERK signaling pathway are described.

### RAF mutations

As mentioned above, V-Raf serves as a ser/thr protein kinase, of which C-Raf is rarely mutated in tumors [246]. The role of RAF was identified until the observation of BRAF(V600E). Alterations in BRAF are frequently observed, while the frequency of C-Raf, A-Raf, and KRAS mutations is much lower than that of BRAF [247]. BRAF mutations frequently occur in melanoma, thyroid adenocarcinoma, colon carcinoma, gallbladder carcinoma, ovary cancer, and lung carcinoma. RAF mutations in tumors are frequent in their specific regions of protein and can be separated into several subgroups according to the way they trigger the pathway [248]. Multiple groups of RAF alterations have been observed. The abnormal activation of RAF mimics the phosphorylation of the activation, which belongs to the first group [248]. The second group promoted RAF to relieve the autoinhibitory effect. The third group had no effect on its activity. They promoted the RAF/MEK/ERK signaling pathway via their wild-type counterparts. The first group of RAF mutations occurred in V600 mutations, including V600D/E/R/K, and V600E was the most common site based on the Catalog of Somatic Mutations in Cancer (COSMIC) database. The activity of B-Raf does not rely on RAS [249]. The second group of RAF mutations are commonly observed in the activation loop and Gly-rich loop, which break the autoinhibitory status and consist of an activation loop and Gly-rich loop. Class II RAF mutations could work as medium kinase activity and contribute to dimer formation. The mutations also triggered dimer ERK activation. Third group RAF mutations existed in the Gly-rich loop, the DFG motif, the catalytic loop, or the C-spine. RAF mutations lead to the inactivation of some kinases, and transactivating normal RAF can activate ERK by promoting dimerization affinity.

B-Raf is considered the primary event but is not enough for tumor formation. BRAF mutations may lead to abnormal signaling activation and protein overexpression, which contribute to cell cycle blockade [250]. In hematologic malignancies, including AMLs and acute lymphocytic leukemias (ALLs), the constitutive abnormal activation of the signaling pathway was observed without any distinct alterations [251]. Unrecognized mutations exist in the signals of the pathway, and some kinase deficiency or excess may promote the activation of the pathway. Furthermore, ERK overexpression is significantly associated with worse survival in AML and ALL patients [252]. RAF and MEK inhibitors are promising agents in some pathological subgroups of AML and ALL patients. Mutations in the signaling pathway need further exploration [253].

### MEK and ERK mutations

MEK and ERK mutations are not frequently observed in tumors. Furthermore, MEK and ERK mutations did not co-occur with RAF mutations, which may demonstrate that the role of MEK and ERK mutations in neoplasms was not similar to that of RAF [245]. MEK mutations were correlated with resistance to RAF and MEK small molecule inhibitors [254, 255]. MEK deficiency facilitates resistance to RAF inhibitors by promoting ERK signaling flux. MEK mutations induced the activation of MEK signaling by disturbing its activity mediated by regulatory helix A. The activation of MEK signaling could also be turned on by MEK mutations by promoting MEK homodimerization [254, 256]. In consideration of the different mechanisms of the two types of MEK mutations and MEK signaling activation, MEK mutations presented distinct sensitivities to MEK inhibitors in animal models and clinical trials, which were similar to RAF mutations.

## RAF/MEK/ERK pathway inhibitors

**Fig. 3:**
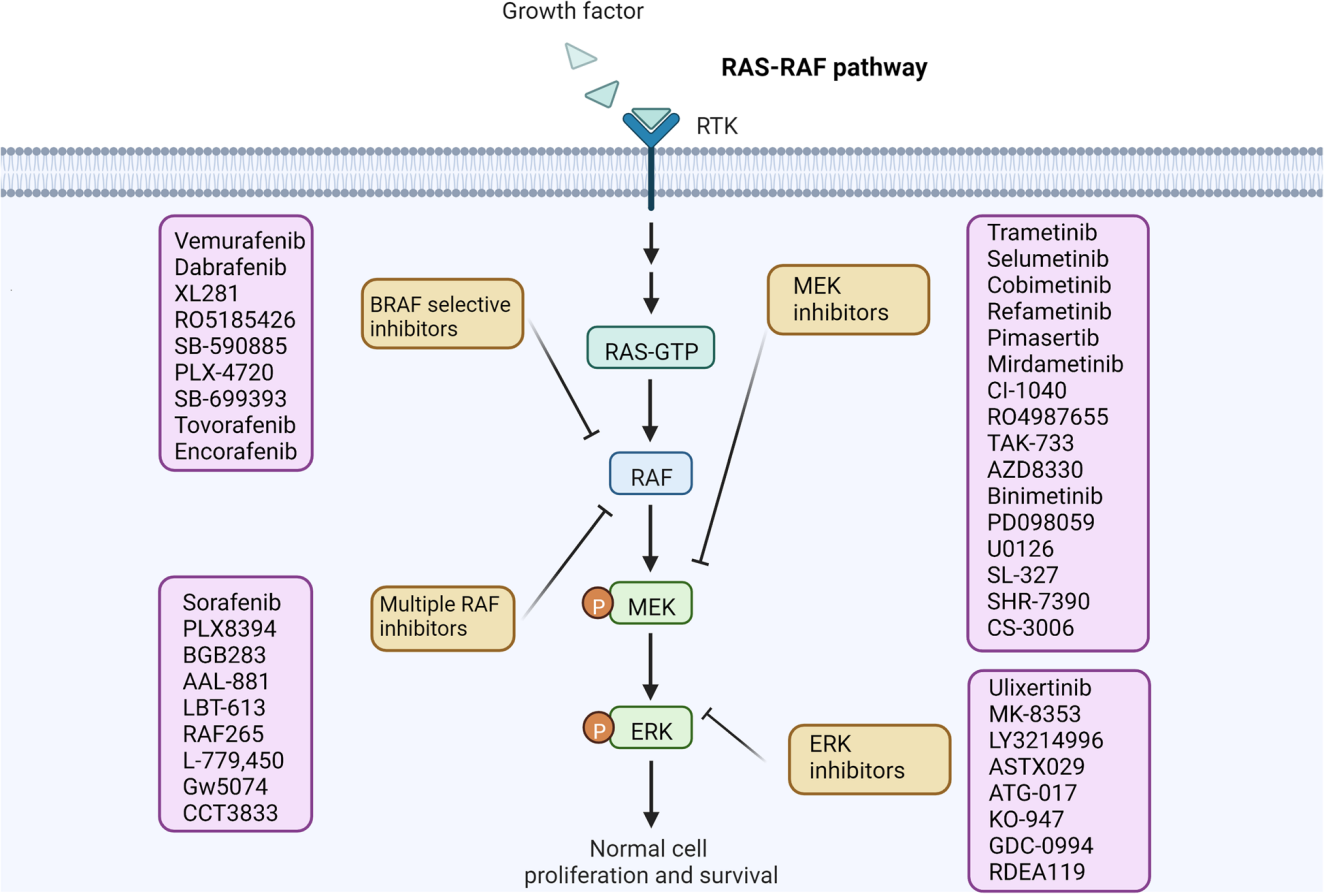
Comprehensive understanding and agent direction for targeting the RAF/MEK/ERK signaling pathway in cancer treatment

The RAF/MEK/ERK signaling pathway is critical in tumor cell growth, cell apoptosis, cell differentiation and cellular metabolism [257]. GTP-bound Ras can recruit RAF to the plasma membrane and facilitate only substrates, Mek1 and Mek2 phosphorylation [258]. Mek1 and Mek2 promote Erk1 and Erk2 activation, enabling Erk1 and Erk2 to phosphorylate more than 70 substrates containing nuclear transcription factors [259]. Based on the mechanisms and functions of the RAF/MEK/ERK signaling pathway, small molecular inhibitors targeting the signaling pathway have demonstrated excellent treatment effects in cancer patients (Fig. 3) [260]. Inhibitors of the RAF/MEK/ERK signaling pathway are potential agents in tumor therapy, and many compounds have been developed in clinical trials and preclinical studies [257]. Tour kinds RAF and MEK inhibitors have been approved. No ERK inhibitors have been approved.

### RAF and MEK inhibitors

A series of RAF inhibitors are undergonig clinicals, and some are in preclinical research (Table 3). Generally, RAF inhibitors demonstrated a higher ORR in clinical cancer patients than MEK inhibitors, which may be associated with the extensive effect of RAF inhibitors that inhibit ERK activation [261]. In contrast, MEK inhibitors inhibited MEK in cancer or normal cells rather than other targets. Many inhibitors were initially considered as single target drugs, but with the pharmacodynamics of drug development, their effects on multiple targets were found [226]. This observation has no influence on their effect on tumor inhibition.

**Table 3:** Inhibitors of RAF/MEK/ERK

Inhibitors/natural products	Target	Studies found in Clinical Trials.gov	Sponsor (Company)	Conditions	Study stage
Sorafenib (BAY 43-9006)	BRaf, VEGFR-2, VEGFR-3, PDGFR b, Flt-3, and c-KIT	916	Bayer	Pancreatic Cancer, Acute Myeloid Leukemia, Unresectable Hepatocellular Carcinoma, Metastatic Renal Cell Carcinoma, Thyroid Cancer, Colorectal Cancer Metastatic, Neuroblastoma, NSCLC, Bladder Cancer, Prostate Cancer, Sarcoma	Approved
PLX8394	BRafV600E, WT BRaf, CRaf	1	Fore Biotherapeutics	Melanoma, Thyroid Cancer, and Colorectal Cancer	I, II
BGB283	BRafV600E,EGFR	1	BeiGene	solid tumor	I, II
AAL-881	Raf		Novartis		Preclinical
LBT-613	Raf		Novartis		Preclinical
RAF265	B-Raf, Raf-1 (cRaf), A-Raf, B RafV600E, VEGFR-2	2	Novartis	Metastatic Melanoma	I
L-779, 450	Raf		Merck		Preclinical
Gw5074	Raf-1 (cRaf)		GlaxoSmithKline		Preclinical
CCT3833 (BAL3833)	Raf	1	Royal Marsden NHS Foundation Trust	Melanoma	I
RO5126766	Raf/MEK	5	Plexxikon/Roche	Advanced NSCLC, Multiple Myeloma	I
RO5185426	Raf, BRafV600E	43	Plexxikon/Roche	Malignant Melanoma, Multiple Myeloma, Unresectable Papillary Thyroid Cancer, Pediatric Recurrent/Refractory BRAFV600E-mutant Gliomas, and NSCLC	I, II, III
SB-699,393	BRaf		GlaxoSmithKline		Preclinical
PLX-4032 (Zelboraf/RG7204/Vemurafenib)	Raf, BRafV600E	173	Plexxikon/Roche	Malignant Melanoma, Colorectal Carcinoma, Hairy Cell Leukemia, and Thyroid Cancer	Approved
GSK2118436 (Dabrafenib/Tafinlar)	BRaf	160	GlaxoSmithKline	Papillary Thyroid Carcinoma, Melanoma, NSCLC, Metastatic Colorectal Cancer, Glioblastoma, and Metastatic Non-clear Cell Renal Cell Carcinoma	Approved
BMS-908,662/XL281	BRaf	2	Exelixis/Bristol Myers Squibb	Colorectal Cancer, and Melanoma	I
TAK-580 (Tovorafenib/DAY101 /MLN2480)	BRaf	6	Takeda	Advanced Nonhematologic Malignancies, and Melanoma	I
Encorafenib (Braftovi /LGX818)	BRaf	73	Pfizer	Melanoma, Locally Advanced Pancreatic Carcinoma, Hairy Cell Leukemia, NSCLC, and Metastatic Colorectal Cancer	Approved
SB-590,885	Raf, BRafV600E		GlaxoSmithKline		Preclinical
PLX-4720	Raf, BRafV600E		Plexxikon/Roche		Preclinical
Trametinib (GSK1120212/Mekinist/JTP74057)	MEK1/2	253	GlaxoSmithKline	Central Nervous System Glioma, Non-small Cell Lung Cancer, Colon Cancer, Advanced Lymphoma, Metastatic Pancreatic Carcinoma, Thyroid Gland Anaplastic Carcinoma, Cholangiocarcinoma, Metastatic Triple-Negative Breast Cancer, and Hepatocellular Cancer	Approved
selumetinib (AZD6244/Arry-142,886)	MEK1/2	127	AstraZeneca	Melanoma, Liver cancer, Pancreatic Carcinoma, Colorectal Cancer, NSCLC, Breast Cancer, Acute Lymphoblastic Leukemia, Sarcoma, Recurrent Adult Diffuse Large Cell Lymphoma, and Thyroid Gland Carcinoma	Approved
GDC-0973 (Cobimetinib/Cotellic/XL518)	MEK1/2	116	Genentech, Pfizer	Malignant Melanoma, NSCLC, Relapsed or Refractory AML, Breast Cancer, Gallbladder Cancer, Ovarian Cancer, and Colorectal Cancer	Approved
Refametinib(BAY86-9766/RDEA119)	MEK1/2	10	Bayer	Advanced Pancreatic Cancer, and Hepatocellular Carcinoma	I, II
Pimasertib (AS703026/MSC1936369B)	MEK1/2	14	Merck	Mutated Cutaneous Melanoma, Ovarian Cancer, Hepatocellular Carcinoma, Metastatic Colorectal Cancer, and Pancreatic Adenocarcinoma	I, II
Mirdametinib (PD-0325901)	MEK1/2	12	Pfizer	Breast cancer, Colorectal cancer, NSCLC, Melanoma, Colorectal Cancer, and Low-Grade Glioma	I, II
CI-1040 (PD184352)	MEK1, MKK5	2	Pfizer	Colorectal Neoplasms, Breast Neoplasms, NSCLC, and Pancreatic Neoplasms	I, II
RO4987655	MEK1/2	1	Hoffmann-La Roche	Neoplasms	I, II
TAK-733	MEK1/2	1	Millennium Pharmaceuticals	Advanced Metastatic Melanoma	I
AZD8330	MEK1/2	1	AstraZeneca	Solid tumors	I
MEK162(ARRY-483,162/Binimetinib/Mektovi)	MEK1/2	124	Pfizer	Advanced Gastrointestinal Stromal Tumor, Metastatic Colorectal Cancer, Lung cancer, Biliary Tract Cancer, and Leukemia,	Approved
PD098059	MEK1/2		Parke-Davis/Pfizer	Advanced Hematological, and advanced solid cancers	Preclinical
U0126	MEK1/2		DuPont Pharmaceuticals		Preclinical
SL-327	MEK1/2		DuPont Pharmaceuticals		Preclinical
SHR-7390	MEK1/2	5	Jiangsu HengRui Medicine	Metastatic Hormone-Resistant Prostate Cancer, and Advanced Breast Cancer	I
CS-3006	MEK1/2	2	CStone Pharmaceuticals	Locally Advanced or Metastatic Solid Tumors	I
Ulixertinib (BVD-523)	ERK1/2	13	BioMed Valley Discoveries	Gastrointestinal Neoplasms,Non-Hodgkin Lymphoma, Pancreatic Cancer, Uveal Melanoma,AML	I, II
MK-8353	ERK1/2	3	Merck Sharp & Dohme LLC	Advanced Solid Tumors	I
LY3214996	ERK1/2	11	Lustgarten Foundation	AML, Unresectable or Metastatic Colorectal Cancer, Recurrent Glioblastoma,Pancreatic Cancer, and CLL	I, II
ASTX029	ERK1/2	2	Astex Pharmaceuticals	Advanced Solid Tumors	I, II
ATG-017	ERK1/2	1	Antengene Corporation	Advanced Solid Tumors, and Hematological Malignancies	I
KO-947	ERK1/2	1	Kura Oncology	Non-Hematological Malignancies	I
GDC-0994	ERK1/2	2	Array BioPharma	Locally Advanced or Metastatic Solid Tumors	I

*CLL* Chronic lymphocytic leukemia, *SLL* Small Lymphocytic Lymphoma, *FL* Follicular Lymphoma, *HNSCC* Head and Neck Squamous Cell Carcinoma, *NSCLC* Non Small Cell Lung Cancer, *CNS* Central Nervous System, *AML *Acute Myeloid Leukemia, *SCLC * Small Cell Lung Cancer

GSK2118436 (dabrafenib, Tafinlar), PLX-4032 (zelboraf, RG7204, and vemurafenib), encorafenib (Braftovi and LGX818), and sorafenib (BAY 43-9006) have been approved. It has been reported that sorafenib is approved for treating RCC and HC, while it is not a pure RAF inhibitor [262, 263]. Sorafenib has been generally considered a first-line treatment method for patients with advanced HC. Early identification of patients who would benefit from sorafenib is essential because many HC patients do not benefit from sorafenib treatment and experience intolerant toxicity [264, 265]. In phase 3 studies in patients treated with sorafenib, more serious AEs and fatal AEs were observed in patients receiving sorafenib plus nontargeted chemotherapy than in those treated with placebo or nontargeted chemotherapy [265, 266]. MEK inhibitors and other inhibitors have been used in treating advanced HC, but they have shown better effects than sorafenib [267]. It was suggested that sorafenib is a multiple target inhibitor that could suppress RAF and other targets [268].

PLX-4720 and PLX-4032 (vemurafenib and RG7204) are two inhibitors produced by Plexxikon/Roche [269]. PLX-4720 is a specific mutant B-Raf inhibitor that has not been researched in clinical trials [269, 270]. PLX-4032 was identified as a potential and selected B-Raf inhibitor of BRAF mutation signaling in 2010 and was approved by the FDA in 2017 [271]. PLX4032 showed a certain effect in melanoma patients with mutant B-RAF in phase 1–3 trials. Overall survival and progression-free survival were also prolonged in untreated melanoma patients [272]. The disease-free survival of melanoma patients was 7 months, which could be improved by PLX-4032 treatment [273]. Approximately 30% of patients treated with PLX-4032 maintained a stable disease status. The results showed that both intrinsic and acquired resistance could influence the clinical efficacy of PLX4032. Overall, the outcomes demonstrated that to prolong the survival of mutant B-RAF melanomas, more efficient small molecule inhibitors are essential. It is vital to improve PLX4032 activity to increase the clinical response and recognize the mechanisms of BRAF mutation in tumors. PLX-4720 is designed as a highly selective BRAF mutation inhibitor that can distinguish between mutant and wild-type proteins [269, 270]. Preclinically, PLX-4720 is efficient in suppressing the progression of colorectal cancer cells and melanoma cells with the V600E mutation. This in tumor cells was considered to be related to more aggressive activity and poor survival. In cellular assays, the IC50 for PLX-4720 in BRAF mutations is significantly lower than that in wild-type patients [274]. In the cell lines, the BRAF status could be detected. The IC50 of PXL-4720 was approximately 100 lower than that of sorafenib in malignant meningioma and colorectal neoplasms with the BRAF V600E mutation. In RAS mutations without BRAF mutations in cell lines, the IC50 of PLX-4720 was similar to that of sorafenib in colorectal cancers and NSCLC. PLX-4720 is a potential agent in patients with B-Raf mutations [274].

Trametinib (GSK1120212, Mekinist, and JTP 74,057) and cobimetinib (GDC-0973, Cotellic, and XL518) are approved for tumors with the BRAF V600E mutation and could be used as monotherapy or plus other therapies [275]. These inhibitors had a high specificity and could strongly suppress the MEK signaling pathway in tumor and normal cells, which do not block ATP.

Trametinib was approved in 2017 for treating unresectable BRAF V600E or V600K mutation melanoma patients [276]. The effect of trametinib is undergoing clinical trials of other neoplasms [277]. The median overall survival of locally recurrent pancreatic cancer patients treated with stereotactic body radiotherapy (SBRT) plus pembrolizumab and trametinib was 24.9 months compared with 22.4 months in patients treated with SBRT plus gemcitabine [278]. There were no therapy-related deaths reported. Compared with SBRT plus gemcitabine regimens, less neutropenia or thrombocytopenia was found in SBRT plus pembrolizumab and trametinib. SBRT plus pembrolizumab and trametinib may be a potential approach to patients with relapsed pancreatic carcinoma. Selumetinib (AZD6244, Arry-142,886) and MEK162/ARRY-483,162 (binimetinib and Mektovi) are two other agents approved by the FDA in 2016 and 2020, respectively [279, 280]. In addition to these small molecule inhibitors, dozens of MEK inhibitors are in the clinic, and some are in preclinical trials, such as refametinib (BAY86-9766, RDEA119), pimasertib (AS703026, MSC1936369B), mirdametinib (PD-0325901), CI-1040 (PD184352) and RO4987655 (Table 3).

### ERK inhibitors

Some agents targeting the terminal kinase ERK have been developed, but most have been in preclinical trials, and few have been developed in clinical trials. Their effect in RAF or MEK-mutated tumors is unclear [281, 282]. Similar to MEK inhibitors, ERK inhibitors have no selectivity in tumor cells and normal cells, which may result in severe toxicity and unfavorable survival. ERK inhibitors combined with RAF inhibitors, as synergetic agents, may have amazing antitumor activity [281, 282]. In MAPK-related tumors with BRAF mutations, BRAF and MEK inhibitors improved survival. Furthermore, resistance to MAPK inhibitors results in abnormal regulation of ERK, and the phosphorylation of ERK increases [283]. The application of an ERK1/2 kinase inhibitor could relieve MAPK inhibitor resistance in tumor cells with BRAF mutations.

Overall, the application of RAF/MEK/ERK inhibitors contributes to targeted therapy for tumors, and the effect of RAF/MEK/ERK inhibitors facilitates the understanding of RAF mutations in tumor therapy. Comprehensive investigation of the relationship of RAF/MEK/ERK mutations and wild-type counterparts provides new insight into novel allosteric targets to help the treatment of neoplasms.

## Cross-talk between the PI3K/AKT/mTOR and RAF/MEK/ERK pathways

Apoptosis induced by the RAF/MEK/ERK and PI3K/AKT/mTOR signaling pathways is regulated by key factors that are regulated by the phosphorylation of ERK or AKT [284]. HER2 overexpression results in abnormal activation of the RAF/MEK/ERK and PI3K/AKT/mTOR signaling pathways in breast cancer [257]. Furthermore, class III RTK uptake in upstream clouds also leads to the activation of the two pathways in AML. The suppressor p53 plays a vital role in both the PI3K/AKT/mTOR and RAF/MEK/ERK pathways [251]. The activity of p53 could be mediated by the PI3K/AKT/mTOR and RAF/MEK/ERK pathways. Approximately 90% BRAF mutations, 45% PTEN phosphatase-related gene depletion and 45% AKT amplification are found in melanomas. All these mutations contributed to the activation of AKT, which indicated poor survival. In addition to these mutations, signaling from RAS and the cell surface also leads to PI3K phosphorylation, contributing to AKT activation. Cosuppression of the RAF/MEK/ERK and PI3K/AKT/mTOR signaling pathways by RAF plus AKT inhibitors or mTOR inhibitors led to synergistic inhibition [285]. Dactolisib is an approved dual PI3K/mTOR inhibitor, and selumetinib is an approved MEK inhibitor [286]. Dactolisib combined with selumetinib resulted in the synergistic action of lung carcinoma with KRAS and PIK3CA mutations. The treatment response of a RAF kinase, sorafenib, could be enhanced by the administration of mTOR inhibitors in a hepatoma carcinoma cell tumor xenograft model [287, 288]. Dactolisib improved the treatment effect of Raf inhibitors in differentiated and medullary thyroid cancers.

Cell growth and protein production were suppressed by inhibiting MEK and mTOR simultaneously in human NSCLC cells [289]. Inhibiting both MEK and mTOR suppressed ribosomal biogenesis and was related to obstruction of translation [290, 291]. The results were identified in a mouse xenograft model. These results indicated that inhibiting both pathways could not only promote cell apoptosis but also converge to mediate the production of proteins. ERK mediates the initial translation activity via Mnk1/2 and p90Rsk phosphorylation [285]. Phosphorylated 4EBP1 is inhibited in BRAF-mutant cells [292]. AKT and mTOR were suppressed, and the activity of 4EBP1 was inhibited. It is clear that the translation of some mRNAs was promoted in cells with BRAF mutations. The production of certain proteins may result in synergistic responses. More potent mechanisms need further exploration.

## Improving the effect of targeting the RAF/MEK/ERK and PI3K/AKT/mTOR pathways by simultaneous treatment

With the development of RAF/MEK/ERK and PI3K/AKT/mTOR pathways, combinations of RAF and PI3K/AKT/mTOR or MEK and PI3K/AKT/mTOR inhibitors are undergoing clinical trials. The combination application of the agents in advanced solid neoplasms was well tolerated and was a single agent. The clinical response and long-term survival were observed. The signals from the RAF/MEK/ERK signaling pathway negated PI3K/AKT/mTOR activation. Both signaling pathways are regulated by RAS and RTK on the surface and stimulated by second messengers. ERK phosphorylation was promoted by mTORC1 dephosphorylation of its residues in malignant mammary patients after therapy. The RAF/MEK/ERK signaling pathway was activated in patients with TNBC treated with buparlisib. The MEK inhibitor combined with buparlisib demonstrated a superior antitumor effect, indicating that inhibiting the PI3K/AKT/mTOR and MAPK/MEK/ERK pathways could have synergistic action [293, 294]. In contrast, PI3K inhibitors also play vital roles in RAF/MEK/ERK signaling pathway dysregulation. The alterations in PTEN may upregulate AKT activity, which attenuates apoptosis-induced BRAF inhibition. However, PI3K inhibitors plus BRAF inhibitors demonstrated promising effects compared with single inhibitor treatment [295, 296]. An AKT inhibitor (GSK2141795) and MEK inhibitor (GSK1120212) combination have been used in cancer patients. The study was completed in 2017, but no results were posted. The primary clinical response to PI3K/mTOR inhibitors (gedatolisib) plus MEK inhibitor (PD-0325901) was presented in ovarian carcinoma and endometrial carcinoma. According to the potential effect of the agents combination, MEK1/2 inhibitor (MEK162) plus the PI3K/mTOR dual inhibitor (BEZ-235) was also evaluated in selected advanced solid cancer patients, which included EGFR mutant NSCLC patients with response to EGFR inhibitors, TNBC, pancreatic carcinoma, colon carcinoma and melanoma with KRAS, NRAS, and/or BRAF mutations patients. The combination of a dual PI3K/mTOR inhibitor (BEZ-235) and a mTOR inhibitor (RAD-001) was evaluated in a phase 1 clinical trial. A MEK inhibitor (MSC1936369B) combined with a PI3K/mTOR inhibitor (SAR245409) demonstrated severe toxicity and poor clinical response [169].

## Conclusion

Both the PI3K/AKT/mTOR and RAF/MEK/ERK pathways are frequently involved in cancer therapy. Both PI3K/AKT/mTOR and RAF/MEK/ERK can be regulated by p53, which contributes to cell proliferation, drug resistance, cell cycle progression and tumor metastasis [71]. Many oncogenes are trapped by retroviruses, including RAS, PI3K, AKT, Src, Abl, RAF, Fos, and Jun [229]. These genes in the two pathways have been found to be frequently and aberrantly mediated. Alternations in upstream receptors serving to PI3K/AKT/mTOR and RAF/MEK/ERK activation have also been widely discussed in the development of tumors [257]. Mutations upstream result in multiple abnormal downstream activation. These factors lead to the complicated treatment of tumors and targeted drug failure because single-target small molecular inhibitors may not suppress additional downstream factor activity. In addition, more subsequent mutations may be acquired, which contribute to resistance and tumor cell anti-apoptosis. The results indicated that mutations and additional activation should be taken into consideration during the development of inhibitors targeting these genes, which may relieve drug resistance. Although the mechanisms of the PI3K/AKT/mTOR and RAF/MEK/ERK pathways are different, the two pathways share many downstream targets that may promote cell proliferation and facilitate drug resistance in an alternative way. In addition to mutations, abnormal activation is another reason for tumor development and drug resistance. This reason also leads to limited effectiveness in patients treated with inhibitors.

In the RAF/MEK/ERK pathway, MEK inhibitors initially demonstrated the most specificity [230]. Some neoplasm patients benefit from MEK inhibitors, but some do not. MEK activation may not be the only target responsible for the disease. This limited the efficiency of MEK inhibitors. Therefore, the combination of MEK inhibitors with chemotherapy or radiotherapy may demonstrate promising effects. MEK inhibitor monotherapy promoted cell apoptosis and induced AEs. Therefore, the tolerance of the combination therapy deserves more attention [245]. A similar situation was observed in selective RAF inhibitors, while some Raf inhibitors have multiple targets. In addition to suppressing BRAF, sorafenib also inhibited VEGFR and PDGFR to block the cell cycle, promote cell apoptosis and relieve drug resistance. This promiscuous nature of sorafenib makes it effective in certain cancers. In addition to combination with chemotherapy and radiotherapy, dual inhibitors are another hot spot, such as RAF and PI3K inhibitors. These targets are upstream of pathways, and mutations are frequent, which may result in exciting effectiveness. The trouble is that RAS activation leads to Raf-1 activation, which may have a limited response to the inhibitors [230]. Therefore, inhibitors in combination with traditional drug/physical treatment are still widely researched. Likewise, dual PI3K and mTOR inhibitors may be a potential antitumor approach compared with either PI3K or mTOR inhibitors. Dozens of dual PI3K and mTOR inhibitors are undergoing preclinical assays and clinical trials. The effectiveness and toxicity are expected.

Thus, the concepts of the two pathways were summarized. First, with the wide understanding of the PI3K/AKT/mTOR and RAF/MEK/ERK pathways, hundreds of inhibitors have been developed. Some of them demonstrated promising effects and moderate toxicity in particular cancers, while some were abolished for certain reasons. Some potential mechanisms that are unknown with further exploration are essential. Second, crosstalk between PI3K/AKT/mTOR and RAF/MEK/ERK is complex and not fully discussed. The targets in the two pathways dependent or independently regulated cell proliferation, apoptosis, and other characteristics. Therefore, multiple target inhibitors are desirable. Third, some inhibitors were abolished, and some clinical trials were withdrawn because of their toxicity and AEs. How to solve this is another crucial question.

The PI3K/AKT/mTOR and RAF/MEK/ERK pathways are intriguing aspects of human cancer therapy and are two complex cascades containing many targets. Further studies on reducing toxicity and improving effectiveness should be conducted. Accumulated studies on these two pathways will provide new hope for cancer patients.
